# Phytochemical, Antimicrobial, and Antioxidant Activity of Different Extracts from Frozen, Freeze-Dried, and Oven-Dried Jostaberries Grown in Moldova

**DOI:** 10.3390/antiox13080890

**Published:** 2024-07-23

**Authors:** Viorica Bulgaru, Angela Gurev, Alexei Baerle, Veronica Dragancea, Greta Balan, Daniela Cojocari, Rodica Sturza, Maria-Loredana Soran, Aliona Ghendov-Mosanu

**Affiliations:** 1 Faculty of Food Technology, Technical University of Moldova, 9/9 Studentilor St., MD-2045 Chisinau, Moldova; viorica.bulgaru@tpa.utm.md (V.B.); angela.gurev@chim.utm.md (A.G.); alexei.baerle@chim.utm.md (A.B.); veronica.dragancea@chim.utm.md (V.D.); rodica.sturza@chim.utm.md (R.S.); aliona.mosanu@tpa.utm.md (A.G.-M.); 2 Department of Preventive Medicine, “Nicolae Testemitanu” State University of Medicine and Pharmacy, 165 Stefan cel Mare Blvd., MD-2004 Chisinau, Moldova; greta.balan@usmf.md (G.B.); daniela.cojocari@usmf.md (D.C.); 3 National Institute for Research and Development of Isotopic and Molecular Technologies, 67-103 Donat, 400293 Cluj-Napoca, Romania

**Keywords:** jostaberry, pretreatment, ultrasound-assisted extraction, microwave-assisted extraction, total polyphenol content, total flavonoid content, total anthocyanin, antimicrobial activity, mutual information

## Abstract

In this paper, the qualitative and quantitative profile is evaluated of the bioactive compounds, antioxidant activity (AA), microbiostatic properties, as well as the color parameters of jostaberry extracts, obtained from frozen (FJ), freeze-dried (FDJ), and oven-dried berries (DJ). The optimal extraction conditions by ultrasound-assisted extraction (UAE) and microwave-assisted extraction (MAE) were selected after determination of the total polyphenol content (TPC), total flavonoid content (TFC), total antocyanin content (TA), AA by 2,2-diphenyl-1-picrylhydrazyl-hydrate (DPPH), and the free radical cation 2,2-azinobis-3-ethylbenzothiazoline-6-sulfonates (ABTS). Non-conventional extraction methods are less destructive to anthocyanins, while drying the berries reduced TA, regardless of the extraction method. The oven-drying process reduced the concentration of TA in DJ extracts by 99.4% and of ascorbic acid by 92.42% compared to FJ. AA was influenced by the jostaberry pretreatment methods. The DPPH and ABTS tests recorded values (mg Trolox equivalent/g dry weight) between 17.60 and 35.26 and 35.64 and 109.17 for FJ extracts, between 7.50 and 7.96 and 45.73 and 82.22 for FDJ, as well as between 6.31 and 7.40 and 34.04 and 52.20 for DJ, respectively. The jostaberry pretreatment produced significant changes in all color parameters. Mutual information analysis, applied to determine the influence of ultrasound and microwave durations on TPC, TFC, TA, AA, pH, and color parameters in jostaberry extracts, showed the greatest influence on TA (0.367 bits) and TFC (0.329 bits). The DPPH and ABTS inhibition capacity of all FJ’ extracts had higher values and varied more strongly, depending on pH, heat treatment, and storage time, compared to the AA values of FDJ’ and DJ’ extracts. A significant antimicrobial effect was observed on all bacterial strains studied for FJP. FDJP was more active on *Bacillus cereus*, *Staphylococcus aureus*, and *Escherichia coli*. DJP was more active on *Salmonella* Abony and *Pseudomonas aeruginosa*. The antifungal effect of DJP was stronger compared to FDJP. Jostaberry extracts obtained under different conditions can be used in food production, offering a wide spectrum of red hues.

## 1. Introduction

The jostaberry hybrid (*Ribes × nidigrolaria*) is a fruiting shrub obtained by crossing three original species: the blackcurrant *Ribes nigrum*, the North American coastal black gooseberry *Ribes divaricatum*, and the European gooseberry [1,2]. In Moldova, jostaberries, which combine the phytochemical characteristics and properties of these berry species, began to be widely cultivated after 2011 [3]. Berries, including the Josta hybrid varieties, are sources of notable nutritional value, due to their increased content of biologically active compounds (BACs), such as fiber, carbohydrates, phenolic compounds, vitamins, and minerals [4,5].

Nijolė et al. noted that blackcurrant berry interspecific hybrids accumulated up to 614 mg/100 fresh weight (FW) of total phenolic compounds (TPCs), and the highest content of anthocyanins was determined in the berries of hybrids *Ribes nigrum × Ribes petraeum*, of 522.8 mg/100 g FW [6]. In the different berry species of black currant, red currant, and a cross between black currant and gooseberry (*Ribes nidigrolaria* L. cv. jostaberry) harvested from Turkey, the anthocyanins cyanidin 3,5 di-glucoside were identified, followed by delphinidin 3-rutinoside, cyanidin 3-rutinoside, and delphinidin 3-glucoside. The predominant phenolic acids in all Ribes species were vanillic acid, 2-hydroxybenzoic acid, and protocatechuic acid [7]. Okatan et al. determined an increased content of ascorbic acid, ellagic acid, gallic acid, ferulic acid, caffeic acid, chlorogenic acid, catechins, rutin, quercetin, and others in different species of *Ribes* and jostaberry (*Ribes × nidigrolaria* Rud. Bauer & A. Bauer) [8].

Other research studies have evaluated the potential of a jostaberry variety as a source of natural bioactive compounds characterizing its physicochemical indices, nutraceutical properties, and antioxidant capacity [9]. Mikulic-Petkovsek et al. identified more than 50 different flavonols (quercetin, myricetin, kaempferol, isorhamnetin, syringetin, and laricitrin) in jostaberry. Quercetin and its derivatives were contained in the largest amount (46–100%) among flavonols [10].

Nowadays, there is an increased interest of researchers in berry phytochemicals and their potential as dietary antioxidants in human nutrition [11]. Whether eaten fresh, frozen, freeze-dried, dried, berries offer considerable health benefits [12,13].

In recent years, berry powder and extracts have been used as dietary supplements, as natural additives, and functional food ingredients [13]. Biocompounds from the forest berry, through the effect of inhibiting reactive oxygen species, reduce oxidative stress in the body [14], have an anti-inflammatory [15,16], anticancer [17], antimicrobial, antifungal [18], and antidiabetic action [19], protect the gastrointestinal [16] and cardiovascular system [20], reduce obesity and cholesterol levels [21], and show immunomodulatory and [22] neuroprotective effects [23].

The season of berries is short, and the shelf life is limited due to their perishability and the tendency to develop mold. The processing and storage time of fruits have a negative impact on the content of bioactive compounds and on their antioxidant activity (AA) [10,24].

In modern food technology, the trend is to maximize nutrient retention both during food storage, freezing, drying, and processing [25,26].

Freezing is considered the least destructive preservation technology for phenolic compounds and is recommended as a pretreatment for berries, which have to be transformed into other products [27].

Drying is the most widely used method to take advantage of the nutritional benefits of berries over a long period. The fruits are dried through different processes, such as natural air-drying, oven-drying, vacuum-drying, microwave-drying, and freeze-drying [28,29]. More studies have confirmed that the drying method influences the berries’ physico-chemical characteristics. Treatment with hot air at high temperatures, for a long time, negatively affects the appearance of the fruits and their BAC content [29,30]. Vitamin C and anthocyanins, found in gooseberries, degrade when exposed to heat, oxygen, metal ions, and other factors commonly found in food processing [30].

Vacuum freeze-drying is a process of removing water or other solvents from frozen products by sublimation. Although it is an expensive process and takes longer than other methods of drying, freeze drying offers high-quality biological products, preserving more effectively the biological and sensory properties of the fruit [31]. The results obtained by Wang et al. showed that the wettability, hygroscopic properties and water retention, soluble proteins, ascorbic acid and other nutrients, polyphenols, resveratrol, chlorogenic acid, anthocyanin, and AA (the quenching capacity of the free radical DPPH^•^ (2,2-diphenyl-1-picrylhydrazyl-hydrate) and the free radical cation ABTS^•+^ (2,2-azinobis-(3-ethylbenzothiazoline-6-sulfonates)) of mulberry fruit after vacuum freeze-drying were superior to hot-air-drying [32].

The content and profile of the extractable BAC of fresh and processed berries also depends on the extraction method. Currently, sustainable extraction methods, such as ultrasound-assisted extraction (UAE) and microwave-assisted extraction (MAE), are replacing conventional methods, which are more economical, require less time, energy, and reagents [33]. At the same time, BAC extracts obtained with green solvents are safe and harmless for the consumer [34].

Researchers have stated that UAE reduces the extraction time of polyphenols from pomegranate peel by 87%, and their antioxidant activity is 22% higher, compared to the activity of polyphenols obtained by maceration [35]. Due to the highest recovered amount of total monomeric anthocyanins of 0.152 mg cyanidine-3-glycoside equivalent per gram of dry weight (Cy3GE/g DW), TPC 49.14 mg gallic acid equivalent (GAE)/g DW, and total flavonoid content (TFC) 18.38 mg quercetin equivalent (QE)/g DW in hawthorn extracts, the UAE method was superior to heat and MAE [36].

The use of microwaves shortens the açai extraction time and ensures both a high level of TPCs and strong AA in the extract [37].

Based on the above, the exploitation of the potential of the less-studied natural sources for the food, pharmaceutical, and cosmetic industries, by applying optimal treatment and processing methods, which are environmentally friendly and do not damage the BAC, are the current objectives for researchers.

In this research, the aim was to evaluate the phytochemical, antimicrobial, and antioxidant activity, as well as the color parameters, of frozen, freeze-dried, and oven-dried jostaberries by studying the qualitative and quantitative profile (ultraviolet–visible spectroscopy (UV-Vis), high-performance liquid chromatography (HPLC)) of UAE and MAE extracts obtained from frozen (FJ), freeze-dried (FDJ) and oven-dried (DJ) jostaberry. The specific conditions for the confectionery and fermented dairy products technologies were simulated, varying the temperature, storage time, and pH of the extracts obtained in optimal extraction conditions (UAE, MAE), and their influence on the color parameters and on the AA were followed.

## 2. Materials and Methods

### 2.1. Chemical Materials

The 6-hydroxy-2,5,7,8-tetramethylchromane-2-carboxylic acid (Trolox) (purity ≥ 97%), 2,2-diphenyl-1-picrylhydrazyl-hydrate (DPPH) (≥95%), 2,2-azinobis (3-ethylbenzothiazoline-6-sulfonic acid), and diammonium salt (ABTS) (≥98%) were provided by Alpha Aesar (Haverhill, MA, USA). Aluminum chloride hexahydrate (≥98%) and the standard compounds: gallic acid (GA) (≥97%), rutin (≥94%), quercetin (≥95%), cyanidin-3-*O*-glucoside (≥95%), ascorbic acid (≥95%), malic acid (≥97%), chlorogenic acid (≥97%), caffeic acid (≥97%), and potassium persulfate were purchased from Sigma-Aldrich (St. Louis, MO, USA). Folin–Ciocalteu phenol reagent (2.1 N) was purchased from Chem-Lab NV (Zedelgem, Belgium). Ethanol, n-hexane, methanol, acetic acid, acetonitrile, sodium carbonate, sodium chloride, chlorohydric acid, sodium hydroxide, anhydrous citric acid, sodium citrate, sodium acetate, potassium chloride, 3,5-dinitrobenzoic acid (DNB), cetyltrimethylammonium bromide, and dimethyl sulfoxide (DMSO) were purchased from Chemapol (Prague, Czech Republic). All reagents used in this study were of analytical or chromatographic grade. All spectrophotometric determinations were performed on a spectrophotometer UV-1900 (Shimadzu, Tokyo, Japan).

### 2.2. Biological Material

The jostaberries (*Ribes × nidigrolaria*) were received from a local producer from Cimişlia, located in the south part of Republic of Moldova, coordinates 40°30′0″ N 28°48′0″ E. The jostaberries were harvested at the end of July 2023. The berries were immediately sorted, washed, dried, and thinly frozen at a temperature of −29 ± 1 °C, during 10 h, then packed and stored at a temperature of −19 ± 1 °C. Over one month after freezing storage (at the end of August), the dry matter content of the frozen berries was determined to be 19.76 ± 0.9%. The frozen berries were freeze-dried or dried by a conventional method.

The freeze-drying process was carried out in the installation Christ Gamma 2/16 LSC plus, up to the dry matter of the jostaberry of 92.0 ± 0.7%, at the following conditions: the first stage was conducted at a pressure of 13 Pa, a temperature of −29 ± 1 °C, and during 10 h; the second stage was carried out at a pressure of 13 Pa, a temperature of 19 ± 1 °C, and during 10 h.

The jostaberries were dried by forced convection in a laboratory oven SLW 115 SMART (Pol-Eco Aparatura, Wodzisław Ślaski, Poland) until the final dry matter content was 96.5 ± 0.4% at the temperature of 65 ± 1 °C, during 10 h. The product obtained by freeze-drying and oven-drying was kept in well-closed packages in the dark.

### 2.3. Characterization of Jostaberry—Physicochemical Analysis

The dry matter was determined by measuring the weight loss due to evaporation of water till a constant mass was attained, at 105 ± 1 °C, (AOAC, 2012), using the drying oven SPJ SLN 53 SMART (POL-EKO APARATURA, Wodzisław Śląski, Poland). The fat, protein, and ash content of the samples were determined using the method reported by the AOAC (2012) [38]. The fat content was determined by the Sohxlet method in a SER148 Solvent Extraction Unit (VELP Scientifica, Monza, Italy). The protein content was estimated by determining the amount of total nitrogen content with a conversion factor of 6.25, using the Kjeldahl method in a UDK129 (VELP Scientifica, Monza, Italy) and the ash content was determined by calcining the samples at 550 ± 2 °C in a furnace (Omron E5CC, Snol, Lithuania).

### 2.4. Antimicrobial Activity

#### 2.4.1. Test Organisms

The antibacterial activity of each tested compound was evaluated using six microbial strains, of which two strains were Gram-positive bacteria (*Staphylococcus aureus* ATCC 29213 and *Bacillus cereus* ATCC 9634), three strains of Gram-negative bacteria (*Escherichia coli* ATCC 25922, *Salmonella* Abony ATCC 6017 and *Pseudomonas aeruginosa* ATCC 27853), and a strain of yeast (*Candida albicans* ATCC 10231). Microbial strains were purchased from the American Type Culture Collection.

#### 2.4.2. Preparation of the Inoculum

The microbial strains were subcultured at 35 °C for 24 h on the Mueller–Hinton agar plate. The microbial inoculum was prepared in 5 mL of sterile saline solution corresponding to the 0.5 McFarland turbidity standard, with concentration equivalents to a cell density of about 107–108 cells/mL.

#### 2.4.3. Agar Well Diffusion Method

Antimicrobial activity was determined using the agar well diffusion method, as displayed by Hsouna et al. [39]. Wells with 8 mm diameter were made on Mueller–Hinton agar plates using a sterile metallic cylinder. The previously prepared inoculum was distributed uniformly on the surface of the agar plates, using a sterile cotton swab. Each well was then filled with 100 μL of the compound. The biological material was dissolved in pure DMSO, after which the Muller–Hinton broth was added. The final concentration of DMSO was less than 4%. The plates were left at 4 °C for 2 h to facilitate diffusion of the extracts into the agar [40], then incubated at 36 ± 1 °C for 24 h for bacteria and at 30 ± 1 °C for 48 h for yeasts, under aerobic conditions. Antimicrobial activity was detected by measuring the total zone of inhibition (including the diameter of the wells) after the incubation period. DMSO was used as a negative control. Tests were performed in triplicate.

#### 2.4.4. Determination of Minimum Inhibitory Concentrations (MIC) and Minimum Bactericidal/Fungicidal Concentrations (MBC/MFC)

MIC and MBC/MFC were determined using the microdilution method in Mueller–Hinton broth [41]. Initially, 0.5 mL of pure DMSO and 9.5 mL of Mueller–Hinton broth were added to 2.5 g of homogenized jostaberry (according to the method described in Section 2.5). The obtained preparations (frozen jostaberry (FJP), freeze-dried jostaberry (FDJP), and oven-dried jostaberry (DJP)) were centrifuged at 7000 rpm for 10 min, the supernatant was separated and diluted in Mueller–Hinton broth to obtain final concentrations between 0.781 and 250 mg/mL. Then, 200 μL of preparation or antimicrobial agent was introduced into test tubes, over which 10 μL of bacterial or fungal suspension was added. The last tube containing the components mentioned above, without the addition of the antimicrobial agent, was considered as a positive control. The tube containing tetracycline and miconazole was used as a negative control. Tubes were incubated at 36 ± 1 °C for 24 h for bacterial strains and for 48 h at 30 ± 1 °C for fungal strains. The MIC values were defined as the lowest concentration of compounds which completely inhibited the growth of microorganisms. The MBC and MFC was defined by the first wells with no visible growth and determined by serially subculturing 10 μL on agar plates and incubating for 24 h at 36 ± 1 °C for bacteria and 48 h at 30 ± 1 °C for yeasts [42].

### 2.5. Jostaberry Extract Characterization

A total of 100 g of frozen jostaberries was slightly crushed with a mixer, 1.0 g of the obtained mass was taken and further homogenized in a pestle mortar with the addition of 3 g of quartz sand (of 0.5–1.0 mm). In order to achieve the same extraction conditions and obtain samples with similar homogeneity, 100 g of freeze-dried and oven-dried jostaberries was slightly crushed with a mixer until a sticky mass was obtained, from which 1.0 g of sample was taken and further homogenized in a mortar with 3 g of quartz sand, until the size of the particles (especially the skin) did not exceed 250–300 µm, analogous to the frozen jostaberry samples. The contents of the mortars were transferred to flasks, the volumes were made up to 103 mL with 60% (*v*/*v*) hydroethanolic solution, with a sample/solvent ratio 1:100 (*m*/*v*). The samples were subjected to extraction. To establish the optimal extraction conditions, BAC were extracted by two unconventional methods: UAE with time variation, and MAE with magnetron power and time variation.

The UAE method was performed in the installation ISOLAB (Laborgeräte GmbH, Eschau, Germany), at a frequency of 37 kHz, a temperature of 25 ± 1 °C (the temperature was maintained by adding ice to the ultrasonic bath), for 5, 10, 15, 20, 25, and 30 min.

MAE was performed in a microwave oven (Samsung MS23F301TAS, Beijing, China) at three magnetron power regimes—100, 180, and 300 W. At each regime, the extraction time was 2, 4, and 6 min.

Afterwards, the samples were centrifuged at 7000 rpm for 10 min and the supernatant was collected, filtered through filter paper, and stored in the refrigerator until analysis.

Thus, to determine the optimal extraction conditions, 6 UAE extracts and 9 MAE extracts of frozen jostaberry (FJ), freeze-dried jostaberry (FDJ), and oven-dried jostaberry (DJ) were obtained, in 2 replicates.

To determine the color and antioxidant activity depending on the pH, temperature, and storage time of the extracts, the extracts FJ′, FDJ′, and DJ′ were obtained under the following conditions: a sample/solvent ratio of 1:20 (*m*/*v*), under the optimal conditions: UAE (for 20 min, 37 kHz), and in three MAE regimes (100, 180, and 300 W for 6 min).

#### 2.5.1. Determination of DPPH Free Radical Scavenging Activity

The Trolox equivalent antioxidant capacity (TEAC) assay by reaction with the free radical DPPH^•^, as described in the literature by Paulpriya et al. [43], was applied. The results were expressed in mg Trolox equivalent (TE)/g DW from calibration curve 0–500 µmol/L, (R^2^ = 0.9992) with Trolox.

#### 2.5.2. Determination of ABTS Free Cation-Radical Scavenging Activity

To determine the capacity of a jostaberry extract to capture the free cation-radical ABTS^•+^, in the TEAC assay, the method described in the literature by Arnao et al. was applied [44]. The ABTS solution was produced by the reaction between 7 mM ABTS stock solution with 2.45 mM potassium persulfate (final concentration) for 16 h, in the dark, at room temperature. The ABTS working solution was prepared by diluting the stock solution with ethanol (EtOH) to an absorbance of 0.70 ± 0.02 at 734 nm. After addition of 100 μL of sample or Trolox standard to 3.9 mL of diluted ABTS solution, absorbance was measured at 734 nm after exactly 6 min of incubation at 30 °C. The results were expressed as mg TE/g DW.

#### 2.5.3. Total Phenolic and Flavonoid Content (TPC and TFC)

TPC and TFC were determined spectrophotometrically, by known methods [45] with some modifications [46], using the Folin–Ciocalteu phenol reagent [47] in relation to the calibration curve with gallic acid standard (0–500 mg/L, R^2^ = 0.9977) and expressed in mg GAE/g DW.

The TFC was determined with AlCl_3_·6H_2_O according to the quercetin (0–160 mg/L, R^2^ = 0.9972) and rutin (0–160 mg/L, R^2^ = 0.9991) calibration curve. The results were expressed in mg QE/g DW and mg rutin equivalent (RuE)/g DW.

#### 2.5.4. Total Anthocyanin (TA)

The TA of the jostaberry extracts was determined with a UV-Vis spectrophotometer, by the pH-differential method, described in [48,49].

### 2.6. Color Analysis

The color analysis of jostaberry extracts samples were performed using a Chroma Meter CR-400 (Konica Minolta, Osaka, Japan). The CIELab color scale was used to obtain the lightness (L*), the red–green parameter (a*), and the yellow–blue parameter (b*) values. The chromaticity (C*) and the hue angle (h*) values were calculated according to the following formulas:

C* = √(a*^2^ + b*^2^), (1)

h* = arctan (b*/a*)(2)

### 2.7. Obtaining Jostaberry Extracts to Determine the Influence of pH Values, Processing, and Storage Conditions on the Color Parameters and Antioxidant Activity

Frozen, freeze-dried, and oven-dried jostaberries were extracted by the non-conventional methods described in Section 2.5 (in sample/solvent ratio 1:20, *m*/*v*), in the optimal regime, selected in preliminary research: UAE (time 20 min, 37 kHz), and MAE regimes (100, 180, 300 W for 6 min). The jostaberry extracts FJ′, FDJ′, and DJ′, obtained under optimal conditions, were used to determine the AA by DPPH and ABTS, at different pH values (2.5, 3.5, 4.5), adjusted with citric acid monohydrate, with the samples stored under different regimes: 7 days at 4 °C, 7 days at 25 °C, and 4 h at 35 °C. For all obtained extracts in optimal regimes, the color parameters were determined according to the method described in Section 2.6.

#### 2.7.1. High-Performance Liquid Chromatography with Photo Diode Array (HPLC-PDA) Detection

HPLC-PDA analysis was realized on a “Shimadzu LC 2030C 3D Plus” instrument, using reversed-phase C18-column Phoenomenex (150 mm × 4.6 mm × 4 μm) and 2-phase gradient elution. Phase A: water with 0.1% acetic acid. Phase B: acetonitrile with 0.1% acetic acid. Flow: 5% of Phase B. Flow rate: 0.5 mL/min. Column oven temperature: 30 °C. Gradient scheme, related to Phase B: 0.01 min—5%; 2.00 min—5%; 22.50 min—50%; 23.50 min—90%; 24.00 min—90%; 25.00 min—5%; 30.00 min—5%. Cell temperature: 32.00 °C. Detection range: 200–800 nm. Sampling frequency: 12.5 Hz.

Before being analyzed, jostaberry extracts were filtered through 0.22 μm PES (polyethersulfone) membrane filters.

The chromatographic and statistical parameters of the HPLC-PDA detection of the compounds identified in jostaberry extracts are shown in Table 1.

#### 2.7.2. Quantification of Organic Acids

The total organic acid content in the jostaberry extracts was determined using the capillary electrophoresis method, described by [50,51]. The optimal electrolyte was 10 mmol/L 3,5-dinitrobenzoic acid at a pH of 3.6 containing 0.2 mmol/L cetyltrimethylammonium bromide as a flow inverter. The indirect detection of its UV-Vis absorption was made at 254 nm. The total content of organic acids was expressed in mg/g.

### 2.8. Mathematical Modeling

To determine the influence of UAE and MAE on TPC, TFC, TA, AA (DPPH, ABTS), pH, and color parameters (L*, a*, b*, C* and h*) in jostaberry extracts (FJ, FDJ, DJ) the MATLAB program was used (MathWorks, Inc., Natick, MA, USA). Also, this program was applied to determine the effect of the extraction medium pH and treatment temperature and preservation time on AA (DPPH, ABTS) and color parameters in the jostaberry extracts (FJ′, FDJ′, DJ′) obtained under optimal extraction conditions with the application of ultrasound (UAE-20) and microwaves (MAE 100-6; MAE 180-6; MAE 300-6). The results of mutual information, measured in bits. The higher the bit value, the more pronounced the influence of the extraction methods, pH, and optimal extraction conditions [52].

### 2.9. Statistical Analysis

The results in this research are presented as mean values standard error of the mean for three parallel measurements. The Microsoft Office Excel 2007 program (Microsoft, Redmond, WA, USA) was used for statistical processing. One-way analysis of variance (ANOVA) was performed according to the Tukey test, with a significance level of *p* ≤ 0.05. Statgraphics software Centurion XVI 16.1.17 (Statgraphics Technologies, Inc., The Plains, VA, USA) was used for statistical analysis.

## 3. Results and Discussion

### 3.1. Jostaberry Characteristics

Jostaberries have a low protein and fat content, specific to fruits. The pretreatment of jostaberries by freeze drying and oven-drying, along with the increase in the dry matter content, means that the concentration of the macronutrients takes place at least three or four times (Table 2).

The jostaberry has a chemical composition similar to the plants from which it comes (blackcurrant and gooseberry), in terms of protein and lipid content, but is much richer compared to them in the content of mineral salts, vitamins, especially vitamin C, and other natural biologically active compounds [53].

### 3.2. In Vitro Antimicrobial Activity of Jostaberries

The results of the antimicrobial activity analysis of FJP, FDJP, and DJP (preparations) are shown in Table 3. Initially, it screening was performed to determine the antimicrobial potential of the compounds tested by the well diffusion method. Most of the samples taken in the study illustrated antimicrobial activity on Gram-positive bacteria (*Bacillus cereus*, *Staphylococcus aureus*), Gram-negative bacteria (*Escherichia coli*, *Pseudomonas aeruginosa*, *Salmonella* Abony), and fungi (*Candida albicans*). FJP had no inhibitory effect on *Candida albicans*.

A stronger antimicrobial effect on all studied bacterial strains was observed for the FJP in comparison to the FDJP and DJP; the exception was *Candida albicans*, for which this compound did not show activity. The lowest concentration of MIC and MBC was shown by FJP against *Bacillus cereus* (2.7 mg/mL). FJP showed weaker bactericidal activity on *Salmonella* Abony (21.2 mg/mL) in comparison to FDJP (110.0 mg/mL) and DJP (83.0 mg/mL).

However, FDJP was more active on *Bacillus cereus* (MIC 3.4 mg/mL), *Staphylococcus aureus* (MIC 13.7 mg/mL), and *Escherichia coli* (MIC 13.7 mg/mL), and DJP was more active on *Salmonella* Abony (MIC 41.6 mg/mL) and *Pseudomonas aeruginosa* (MIC 10.4 mg/mL) in comparison to the action of FDJP on these strains (*Salmonella* Abony: MIC 55.0 mg/mL, *Pseudomonas aeruginosa*: MIC 13.7 mg/mL).

Regarding the antifungal effect, DJP (MIC/MBC 83.0/166.0 mg/mL) was more active compared to FDJP (MIC/MBC 110.0/220.0 mg/mL).

The MIC of the tested preparations was higher compared to the MIC of tetracycline and miconazole for most studied species, and in the case of FJP and DJP, the MIC for *Pseudomonas aeruginosa* was lower (10.6 mg/mL; 10.4 mg/mL) compared to the MIC of tetracycline (12.5 mg/mL).

Similar studies were carried out by Trajković et al., who evaluated the antimicrobial activity of lyophilized fruit juice (BCLJ) and waste extract (BCLW) obtained from the blackcurrant [54]. These compounds showed a bacteriostatic effect on Gram-positive bacteria (*Bacillus cereus*, *Enterococcus faecalis*, *Staphylococcus aureus*), except for *Listeria monocytogenes*, for which the extracts were not effective. It was remarked that BCLJ did not inhibit the growth of Gram-negative bacteria.

In a study by Kranz S. et al., blackcurrant juice had a stronger antibacterial effect on several tested Gram-positive bacteria (*Streptococcus mutans*, *Streptococcus gordonii*, *Streptococcus sobrinus*, *Enterococcus faecalis*) and Gram-negative bacteria (*Fusobacterium nucleatum*, *Porphyromonas gingivalis*, *Aggregatibacter actinomycetemcomitans*), compared to other berry juices [19].

A large number of substances responsible for the antimicrobial effect of berry juices have already been identified. In this regard, phenolic substances appear to be the most active components, occurring either as simple molecules such as flavonoids, phenolic acids, or as complex phenolic polymers, for example, tannins [55].

### 3.3. Selection of the Optimal Extraction Regime: Duration of Ultrasound Action, Duration, and Power of Microwaves

According to the bibliographic data, the extracts obtained from plant matrix by non-conventional methods have also a higher content of BAC and show increased AA, compared to extracts obtained by conventional methods [56].

**Figure 1:**
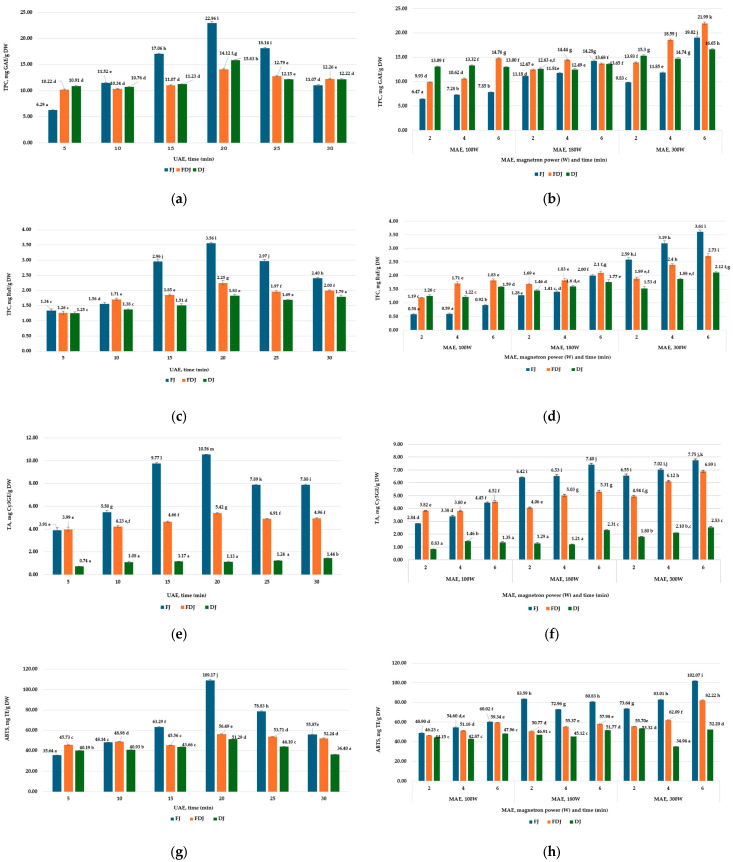
TPC, TFC, TA, and AA in UAE and MAE extracts of FJ, FDJ and DJ (60% EtOH (*v*/*v*), sample/solvent ratio 1:100 (*m*/*v*)). (**a**,**b**) TPC (mg GAE/g DW) in UAE and MAE extracts of FJ, FDJ and DJ; (**c**,**d**) TFC (mg RuE/g DW) in UAE and MAE extracts of FJ, FDJ and DJ; (**e**,**f**) TA (mg Cy3GE/g DW) in UAE and MAE extracts of FJ, FDJ and DJ; (**g**,**h**) AA by ABTS assay in UAE and MAE extracts of FJ, FDJ and DJ. FJ—frozen jostaberry; FDJ—freeze-dried jostaberry; DJ—oven-dried jostaberry; TPC—total polyphenol content; GAE—gallic acid equivalent; DW—dry weight; TFC—total flavonoid content; RuE—rutin equivalent; TA—total anthocyanin; Cy3GE—cyanidin-3-glucoside equivalent; AA—antioxidant activity; TE—Trolox equivalent. The results are presented as the mean of three measurements ± standard deviation (SD). Different letters (^a–m^) designate statistically different results (*p* ≤ 0.05).

In the present study, jostaberries were extracted by eco-friendly methods, in economic mode UAE and MAE, with harmless solvents—60% EtOH solution (*v*/*v*). The optimal extraction conditions were selected after the spectrophotometric determination of TPC (mg GAE/g DW), TFC (mg QE/g DW and mg Ru/g DW), TA (mg Cy3GE/g DW), and determination of DPPH and ABTS AA (Figure 1 and Figure S2) in FJ, FDJ, and DJ extracts.

The results showed that in the case of UAE (37 Hz), the time of ultrasound action in 5, 10, 15, 20, 25, and 30 min influences the TPC, TFC, TA, and AA of jostaberry hydroethanolic extracts (1:100, *w*/*v*). For all the FJ, FDJ, and DJ samples, the highest TPC values were determined for the 20 min experiences (Figure 1 and Figure S2).

The AA of jostaberry extracts in the UAE regime, for 20 min, was also maximal. Increasing the sonication time (30 min) decreased the concentration of BAC and AA in jostaberry extracts (Figure 1 and Figure S2).

Other research has noted that ultrasound action for 40 min reduced the AA of *Terminalia catappa* L. leaves extracts [57]. Also, some bibliographic data showed that the maximum isolation of the anthocyanin pigment is observed after 15 min UAE in 60% ethanol solution at 20 °C [58]. Sady et al. showed that the optimal sonication time for the extraction of black chokeberry anthocyanins was 13 min, and a higher TPC is obtained at a time of 20 min [59]. Jaboticaba skin extracts with hydroalcoholic solution obtained by sonication for 10 min, solid/solvent ratio of 150 *m*/*v* showed the highest ABTS antioxidant capacity (1300 µmol/g DW) and TA (407 mg cyanidin diglucoside equivalent (CyDG)/100 g DW) [34].

MAE, microwave power (100, 180, 300 W) and action time (2, 4, 6 min) also influence the content of BAC and AA in the extracts. When the microwave power and action time increase, the TPC, TFC, TA, and AA values increase, with maximum values for all samples at 300 W, with a duration of 6 min (Figure 1 and Figure S2). Exceeding the optimal time in MAE influenced the solvent ratio, because the evaporation process of ethyl alcohol begins.

The results of Milic et al. [56] showed that MAE (10 min, 600 W, 30% ethanol solution was the most efficient for the extraction of TPC, with 3.41 g GAE/100 g DW from blackcurrants. Also, these parameters allowed a high content of flavonoids to be obtained, of 0.7934 g CE/100 g DW. The DPPH and ABTS antioxidant activity of the hydroethanolic extracts of FJ was the highest in optimal conditions. In the analysis carried out, it was observed that the extraction regime has less influence on the DPPH values of the FDJ and DJ extracts. It is probably explained by the reduced content of vitamins, flavonoids, including anthocyanins, and other bioactive components, which are destroyed during the drying of the jostaberry.

The pH values determined in the obtained extracts varied slightly from one sample to another (Table S1). For the FJ extracts with the use of UAE, the lowest values were obtained, between 4.04 and 4.21. Higher values, between 4.15 and 4.43, were recorded for FDJ and DJ extracts, especially those obtained by the MAE. This phenomenon can be explained by the decarboxylation reactions of strong organic acids upon heating.

### 3.4. Comparative Elucidation of BAC Content in UAE and MAE Extracts of FJ, FDJ, and DJ Determined by the UV-Vis Spectrophotometric Method

The results obtained from this study demonstrate that the concentration of extractable BAC, especially TPC, TFC, and TA determined in jostaberry extracts, is significantly influenced by the method of berry processing: freezing, freeze-drying and oven-drying, as well as by the extraction technique.

#### 3.4.1. Total Polyphenols

The optimal conditions for obtaining higher concentrations of extractable polyphenols in UAE (37 kHz, 20 min) gave a maximal TPC in FJ, FDJ, and DJ extracts of 22.96, 14.12, and 15.83 mg GAE/g DW, respectively. In the MAE regime, TPC went up with increasing magnetron power and microwave duration, reaching maximum values (300 W, 6 min) in the FJ, FDJ, and DJ extracts of 19.02, 21.99, and 16.65 mg GAE/g DW, respectively, Figure 1a,b. The decrease in TPC with the increase in the duration of the ultrasound action is due to the decrease in the concentration of flavonoids (including anthocyanins) sensitive to oxidation. In this study, the values for the TPC, extracted from jostaberries by UAE and MAE methods, are lower, compared to the bibliographic data. The TPC content presented by Okatan in his work on jostaberry extracts was 1593.92 mg GAE/100 g FW, followed by gooseberry 1223.71 GAE/100 g FW, and in blackcurrant, the lowest 11.36 mg GAE/100 g FW [8]. Karaagak et al. [7] identified a TPC of 1809.24 mg GAE/kg FW in fresh jostaberries, much lower compared to the data obtained in the present work. In other research, TPCs were found between 290 and 2611 mg GAE/100 g FW in gooseberries [60].

#### 3.4.2. Total Flavonoids

According to the recorded data shown in Figure 1c,d and Figure S2a,b, freeze-drying and oven-drying had an effect of decreasing the concentration of extractable flavonoids in the extracts of FDJ and DJ. The TFC was higher in the MAE (300 W, 6 min) extracts of FJ (3.56 mg RuE/g and 1.18 mg QE/g DW). TFC values (mg RuE/g DW and mg QE/g DW) decreased by 24.2% and 17.4% in FDJ extracts, and by 41.3% and 40.2% in DJ, respectively, compared to the frozen josta extracts.

A researcher found a quercetin value of 6.32 and a rutin value of 15.73 mg/100 g FW in gooseberry fruits [61]. The amount of rutin, catechin, and quercetin (mg/100 g FW) was determined by HPLC as the highest in jostaberries (22.29, 264.64 and 7.30), in gooseberries (15.53, 124.64 and 6.16), and these values were lower in blackcurrants (15.59, 11.37 and 2.74) [8].

#### 3.4.3. Total Anthocyanins

The non-conventional extraction methods, applied in this study, are less destructive of anthocyanins. At the same time, the oven-drying and freeze-drying of the berries reduced the TA in the FDJ and DJ extracts (Figure 1e,f). In the UAE extracts (20 min) of FJ, the TA determined by the UV-Vis spectrophotometric method had maximum values of 10.56 mg Cy3GE/g DW. In the freeze-dried berries, TA decreased by 48.7%, and in oven-dried ones by 89.3%, compared to FJ, regardless of the UAE or MAE extraction method.

Sadowska et al. determined a decrease in the concentration of anthocyanins by 10% in frozen berries at −18 °C as compared with the fresh fruits. During lyophilization and air-drying, the losses of anthocyanins as compared with fresh fruits accounted for 82% and 94% [30].

In the MAE extracts (300 W, 6 min) of FJ and FDJ, the TA values reached a maximum of 7.75 and 6.89 mg Cy3GE/g DW, respectively, and in the DJ, the TA decreased to values of 2.53 mg Cy3GE/g DW, Figure 1f.

TA, determined by Okatan in fresh jostaberries using the absorbance values taken by spectrophotometer at different pH ranges, had higher values of 114.80 mg/100 g FW than in gooseberries 84.61 mg/100 g FW and blackcurrants 17.59 mg/100 g FW [8].

### 3.5. HPLC Analysis of the Jostaberry Extracts Profile Obtained under Optimal UAE and MAE Conditions

In the extracts of FJ, FDJ, and DJ, obtained under optimal conditions of UAE (20 min, UAE-20) and MAE (100 W, 180 W, 300 W, 6 min, MAE-100-6, MAE-180-6, MAE-300-6), the TA, ascorbic, chlorogenic, caffeic, citric, and malic acid was determined, Table 4.

#### 3.5.1. Anthocyanins

Anthocyanins were adequately extracted with ultrasound, thus the maximum value of TA, calculated in relation to cyanidin-3-O-glucosides (in HPLC determinations) for the frozen jostaberry extract was 17.15 mg/g DW.

In MAE extraction, with the increase in power from 100 to 300 W, the content of extractable anthocyanins in the hydroalcoholic extracts of FJ and FDJ significantly increases. Maximum TA values were recorded for FJ (7.90 mg Cy3GE/g DW) and FDJ (8.59 mg Cy3GE/g DW). The drying process drastically reduced the TA in DJ. The recorded values were almost unchanged in all UAE and MAE extraction regimes, between 0.09 and 0.11 mg/g DW. In the UAE extracts of DJ, the TA values, determined by HPLC, were 99.4% lower compared to FJ. The increase in temperature and the action of light in oven-drying conditions shift the equilibrium of *anthocyanins* towards the chalcone form, accelerating their destruction. In other research, it was mentioned that the results of HPLC analysis showed that jostaberry fruits are sources of phenolic constituents, such as catechins (95.05 mg/100 g FW) and anthocyanins (31.55 mg/100 g FW) [9].

#### 3.5.2. Ascorbic Acid

In the UAE regime, ascorbic acid has lower values in all extracts compared to the values in the MAE regime. The highest content of ascorbic acid (mg/g DW) was detected in MAE extracts (MAE-300-6) of FJ (7.52) and FDJ (6.79), but less in DJ (0.57). The drying process reduced the concentration of ascorbic acid by 92.42% in DJ compared to FJ.

In other studies, it was shown that in freeze-dried and air-dried fruits compared to fresh fruits, vitamin C losses were 84% and 89% [30].

It was established (Table 4) that the decrease in ascorbic acid concentration is accompanied by an increase in the concentration of anthocyanins to maximum values in the UAE extracts of FJ. It can be assumed that ascorbic acid, through its antioxidant properties, is quickly consumed to capture free radicals formed in the UAE regime, protecting anthocyanins from degradation.

The bibliographic sources mention that the experiences carried out in model systems with added ascorbic acid have attested to the phenomenon of anthocyanins’ accelerated degradation and loss of color [62,63]. Other research shows that the presence of ascorbic acid increases the stability of acylated anthocyanins [64]. Anthocyanins are supposed to be protected by ascorbic acid against enzymatic degradation [65]. It turns out that ascorbic acid in food systems, depending on the conditions, can play the role of both an oxidant and an antioxidant.

Okatan et al. determined the highest content of ascorbic acid 2659.26 in blackcurrant using the HPLC method, in jostaberry 451.66, and in gooseberry 157.91 mg/100 g FW [8].

#### 3.5.3. Chlorogenic Acid

In the present study, the highest content of chlorogenic acid (3-caffeoylquinic acid) in FJ was identified with HPLC, of 2.60 mg/g in UAE-20 extracts and 3.28 mg/g DW in MAE 300-6 extracts. The chlorogenic acid concentration decreased in UAE-20 and MAE 300-6 extracts of FDJ by 31.9% and 25.3%, and in DJ extracts by 66.5% and 60.4%, respectively, compared to FJ. The duration and temperature variations during lyophilization and the increased drying temperature promoted the rapid degradation of chlorogenic acid in the jostaberries.

Other research showed a higher chlorogenic acid content (mg/100 g FW) in fresh jostaberries (42.56) compared to gooseberries (35.20) and lower compared to blackcurrants (56.77) [8].

#### 3.5.4. Caffeic Acid and Rutosides

The content of caffeic acid (3,4-dihydroxycinnamic acid) in FJ extracts is also higher, but with values that do not differ much from those found in FDJ and DJ (Table 4). Caffeic acid was better extracted in the MAE 300-6, reaching values of 0.27 mg/g in FJ and 0.22 mg/g DW in FDJ and DJ.

The research showed that the total content of rutosides is higher in UAE-20 and MAE 300-6 FJ extracts at 1.28 and 1.05 mg/g DW, respectively. In the FDJ and DJ extracts under UAE-20 and MAE 300-6 conditions, the rutoside content decreased by almost 43.0% and by almost 6.7%, respectively. These results suggest that the flavonoids in josta fruit are shown to be heat-sensitive. In jostaberries, the content of rutin (mg/100 g FW) with values of 22.29 was reported, of catechins 164.64, and of quercetin 7.30, determined by the HPLC method [8].

Mikulic-Petkovsek et al. [10] reported that flavonols were contained in small amounts in the Ribes species, with the values falling between 5% and 11% of the total phenolic compounds analyzed, and flavonols between 36.12 and 53.94 mg/kg FW in jostaberries.

According to the data, compared to fresh mulberry fruits, the berries pretreated by freeze-drying processes under vacuum showed a much higher content of TPC, TFC, ascorbic, and neo-chlorogenic acid than those subjected to the oven-drying process [32].

#### 3.5.5. Citric and Malic Acids

Also, jostaberries are a rich source of citric and malic acid, with contents ranging from 0.82 to 1.61 mg/g DW for citric acid in UAE extracts. Higher values were recorded for MAE extracts, with a maximum content of citric acid in FDJ, between 2.04 and 3.63 mg/g DW. In the case of malic acid, the maximum values were obtained for DJ extracts. FJ and FDJ extracts have 2- and 4-fold lower malic acid content, respectively, compared to DJ. Probably, the increased concentration of acids in DJ and FDJ is due to the degradation of macromolecules with the release of organic acids in the process of the action of high/low temperatures.

Okatan et al. determined a citric acid content of 14.84 and malic acid of 13.50 mg/100 g FW in jostaberries [8].

### 3.6. Influence of Freeze Drying and Drying Regime on AA by DPPH and ABTS of Jostaberry Extracts—Correlation between BAC and AA of Extracts

One of the objectives of this research was to determine the influence of freeze-drying, oven-drying, and the extraction method on the AA of BAC extractable from jostaberries. Several bibliographic sources state that the TEAC assay for scavenging the free radical DPPH^•^ and the free-cation radical ABTS^•+^ can be used to compare changes in the same antioxidant during processing or storage [66,67].

#### 3.6.1. TEAC Assay by DPPH

The research showed that the data obtained by DPPH method for the FJ samples varied between 17.60 and 35.26 mg TE/g DW and are comparable to those in the literature [68]. In the extracts of FDJ and DJ, the AA by DPPH varies insignificantly, depending on the extraction method, between 7.50 and 7.96 and 6.31 and 7.40 mg TE/g DW, respectively (Figure S2c,d).

Tsuda et al. determined an AA level varying from 52.5 to 456.2 μmol TE/g DW in the extraction of different fresh blueberry varieties using DPPH. Positive correlations were observed between AA and TPC [68].

In FJ extracts, AA by DPPH increases proportionally (Table S2) with the content of polyphenols (R^2^ = 0.8390), flavonoids (R^2^ = 0.7664), anthocyanins (R^2^ = 0.8041), and chlorogenic acid (R^2^ = 0.7263). In FDJ, the DPPH activity of the extracts correlates with the TFC (R^2^ = 0.9882) and ascorbic acid (R^2^ = 0.7525).

The differences between the AA of jostaberry extracts obtained by non-conventional methods are more due to the freeze-drying and oven-drying methods. Thus, the DPPH radical scavenging capacity of the UAE-20 extracts from FDJ and DJ is 77.42 and 79.01% lower, respectively, similar to the values obtained in the MAE 300-6 regime, which decreased by 77.90 and 82.10%, compared to the values obtained for FJ.

The recorded data (Table S2) demonstrate that the TEAC assay by DPPH recorded underestimated AA values for the FDJ and DJ samples. That phenomenon is due to the process of transforming anthocyanins into chalcones and dihydrochalcones during the drying of berries, as well as to the autoxidation process of ascorbic acid and polyphenols [26,69].

The researchers noted that some dihydrochalcones and flavanones did not react with the DPPH radical in contrast to the ABTS radical, leading to significant differences [70]. The oxidation of phenols to less reactive quinones or non-reactive products removes them from the reaction with DPPH [71]. Therefore, to determine the antioxidant potential of extracts rich in dihydrochalcones or flavanones, it is recommended to apply the ABTS test [66,72].

#### 3.6.2. TEAC Assay by ABTS

The TEAC assay for scavenging the radical-cation ABTS recorded AA values (mg TE/g DW) between 35.64 and 109.17 for FJ extracts, between 45.73 and 82.22 for FDJ and 34.04 and 52.20 for DJ extracts (Figure 1g,h), results compatible with the bibliographic ones [73]. Okatan recorded ABTS values for fresh jostaberry extracts of 125.49 mg TE/100 g FW, higher compared to gooseberries (73.23 mg TE/100 g FW) and blackcurrants (74.43 mg TE/100 g FW) [8]. Kim et al. [74] have determined ABTS values between 65.84 and 449.86 µmol TE/g DW in the dry extracts of various berries.

As can be seen (Table S2), the AA using ABTS in FJ, FDJ, and DJ extracts was correlated with total polyphenol concentration. In all FJ samples, AA also increased proportionally with the concentration of anthocyanins (R^2^ = 0.6749) and chlorogenic acid (R^2^ = 0.7621). AA in FDJ samples correlates well with the concentration of anthocyanins (R^2^ = 0.7604), ascorbic acid (R^2^ = 0.9156), and chlorogenic acid (R^2^ = 0.9930). For DJ extracts, the correlation between AA and BAC content is low, so it follows that the oven-drying regime applied (65 ± 1 °C, for 10 h) had an unfavorable effect on anthocyanins, flavonoids, ascorbic, and chlorogenic acids in jostaberries.

Pretreatment by freeze-drying and oven-drying contributed to the reduction in ABTS inhibition capacity, for the FDJ and DJ extracts obtained by UAE-20 by 48.25 and 53.02%, respectively, and by MAE 300-6 by 19.45 and 48.86%, respectively, compared to the values recorded for FJ.

The results confirm that jostaberry drying, especially by the oven-drying method, induces oxidative decomposition, either by enzymatic means or by thermal degradation of the polyphenols and vitamins responsible for the AA effect.

### 3.7. The Influence of Jostaberry Pretreatment and Extraction Methods on the Color Parameters

Jostaberries are rich in flavonoids, especially anthocyanins, a pigment that play a decisive role in the formation of the color of the extracts, which can later be used in the manufacture of food products. The influence of jostaberry pretreatment and extraction conditions on the CIELab color parameters of the extracts are presented in Table 5.

Jostaberry pretreatment produced significant changes in all color parameters. In the case of L*, the highest values were obtained in FJ extracts. In FDJ and DJ extracts, L* values were not significantly different. The highest a* values were obtained in the FDJ extracts followed by DJ. And in the case of the b* values, the highest were obtained for the DJ extracts. The high C* values in the DJ extracts demonstrated the saturation and purity of the extract colors, and in the case of the FJ extracts, the low C* values denote the presence of a gray shade. The h* values for all jostaberry extracts are in the first trigonometric quadrant. The red tone predominates in the FJ and FDJ extracts, and the orange tone in the DJ ones. The results presented in Table 5 demonstrated that the pretreatment conditions of jostaberries influenced the color of the obtained extracts. Hot oven-drying at 65 °C for 10 h affected the integrity of the cell walls of jostaberries and led to the skin breaking. Structural changes within the cells caused the release of compounds bound to the plant matrix and increased their availability [75]. In the case of FJ, the formation of ice leads to an increase in the volume of the fruit and damages its integrity [76,77].

The results of the influence of the UAE duration (5, 10, 15, 20, 25, and 30 min) on the color parameters of all extracts are presented in Table 5.

In the case of FJ extracts, the L* values are in the range of 67.21 and 79.64, the last one refers to the sample, which was obtained when UAE was applied for 30 min. The lowest a* values were obtained in the extracts when UAE was applied for 30 min (16.6) and the highest at 20 min (27.51). The b* changed essentially, the values being in the range from 1.98 (15 min) to 2.57 (30 min), demonstrating the reduced presence of the yellow tone. The C* values in FJ extracts changed from 16.70 (30 min) to 27.59 (20 min), which demonstrates the presence of the gray shade. The h* values are in the first trigonometric quadrant, the values are in the range 4.34° (20 min)–8.85° (30 min), demonstrating the dominance of the red tone.

In the case of FDJ extracts, the L* values changed from 54.96 (5 min) to 62.00 (30 min), demonstrating the increase in the color intensity of the extracts. The a* showed the highest values (57.52) when applying ultrasound for 20 min and the lowest (41.11) at 30 min. This fact proves the presence of the high content of red pigments in the analyzed extracts. The b* changed in the range from 1.52 (5 min) to 12.55 (30 min), the increase in the yellow tone is attested. The C* values of the FDJ extracts changed from 50.05 (5 min) to 42.98 (30 min), so the obtained samples have a saturated color and a high purity. The h* values are in the first trigonometric quadrant and increase from 1.58° (5 min) to 16.98° (30 min) in which the red color predominates.

In the DJ extracts, treated with ultrasound for 15–30 min, the L* values were in the range 51.41–53.58. The highest value of L* (57.87) had the sample treated for 5 min. The highest values of the a* were determined in DJ extracts treated for 15–25 min, being 46.98–45.06, demonstrating the presence of the red tone. The highest b* values (57.64 and 63.64) were determined in DJ extracts treated with ultrasound for 25 and 30 min, respectively, which denotes the presence of yellow pigments. The high C* values in all the investigated samples showed that the extracts have an intense color without the presence of a gray shade. The h* values were in the first trigonometric quadrant, but in this case the values varied from 39.62° to 56.39°, demonstrating the presence of the reddish orange tone.

The results were obtained of the influence of the duration of microwave application (2, 4, and 6 min) at different magnetron powers (100, 180, and 300 W) on the color parameters of all extracts, Table 5.

In the case of FJ extracts, the L* values changed in the range 62.89 (MAE-180-6)–71.45 (MAE-300-2). The highest a* values were obtained when MAE was applied for 6 min at all magnetron powers: 41.11 (100 W), 34.08 (180 W), and 35.16 (300 W), demonstrating the presence of the red tone. The lowest b* values were determined for the same extraction conditions (6 min): 1.88 (100 W), 2.05 (180 W), and 2.01 (300 W). In the case of reduced C* values, all FJ extracts demonstrated the presence of the gray shade. The h* values denote that they are in the first trigonometric quadrant, in which the red color predominates.

In FDJ extracts, the L* values changed in the range 42.56 (MAE-300-6)–67.40 (MAE-180-2), demonstrating the increase in color intensity. The highest a* values were obtained when MAE was applied for 6 min at all magnetron powers: 67.02 (100 W), 69.49 (180 W), and 60.18 (300 W), showing the presence of red pigments. Increasing the duration of MAE application and magnetron power contributed to the reduction in b* values from 14.48 (MAE-100-2) to 7.06 (MAE-300-6). The high C* values indicate that the extracts obtained at all extraction conditions have a saturated color, without the gray shade. The h* values for all FDJ extracts are in the first trigonometric quadrant, in which the red color predominates.

Analyzing the color parameters for DJ extracts, it was found that the L* values changed in the range 43.11 (MAE-300-6)–64.05 (MAE-100-2), demonstrating the increase in color intensity with the increase in the duration of microwave application and magnetron power. It was also highlighted that the highest increases in the a* were obtained in DJ extracts under the following extraction conditions: 49.13 (MAE-100-6), 58.67 (MAE-180-6), and 48.80 (MAE-300-6). In the case of b*, the values are higher than the a* values, demonstrating the dominance of yellow pigments. The high C* values demonstrate that the extracts have a saturated color without a gray tint. The h* values for all DJ extracts are in the first trigonometric quadrant, in which the brown to orange color predominates.

### 3.8. The Influence of Jostaberry Extract pH on the Color Parameters and AA

The pH value influences the stability of anthocyanidins. At an acidic or basic pH level, the highly conjugated phenolic groups of anthocyanidins have the ability to protonate or deprotonate, leading to a change in electronic distribution. This process affects the absorption wavelength and the observed color [78].

To determine the influence of the processing method, the pH of the medium on the color parameters and on the AA, the FJ′, FDJ′, and DJ′ extracts were obtained by the methods described in Section 2.5, but only under the optimal conditions. The extracts of FJ′, FDJ′, and DJ′ were used for the determination of AA by DPPH and ABTS, at different pH values (2.5, 3.5, 4.5).

It was demonstrated that at pH 2.5 the lowest values of L* were obtained, which vary from 33.31 (FDJ′, UAE-20) to 59.29 (DJ′, MAE 300-6) demonstrating the color intensity of the extracts, and the highest values of L* were obtained at a pH of 4.5, ranging from 42.74 (FDJ′, MAE-300-6) to 72.86 (DJ′, MAE 100-6). The analysis of the a* demonstrated that the highest values were obtained at a pH of 2.5, followed by a pH of 3.5 and 4.5. In the case of b*, it was found that the highest values were obtained in DJ′ extracts, showing the presence of yellow pigments. It was found that at pH 2.5, the C* values are the highest in FJ′ and FDJ′ extracts, which denotes the absence of the gray shade. All h* values obtained at pH levels of 2.5, 3.5, and 4.5 are in the first trigonometric quadrant. For FJ′ and FDJ extracts, the red color predominates and for DJ′—the brown color (Table S3).

**Figure 2:**
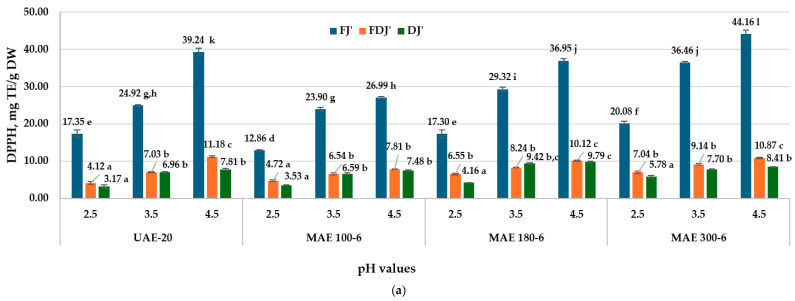

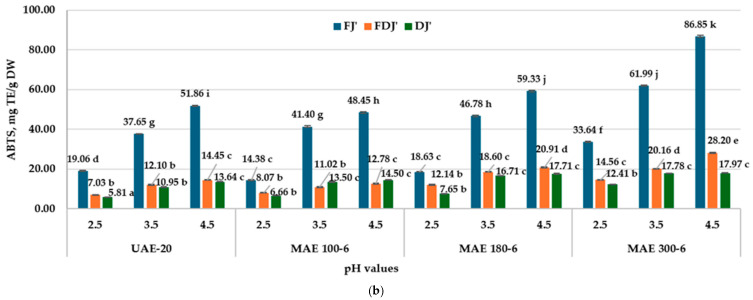
The AA by DPPH (**a**) and ABTS (**b**) in jostaberry UAE and MAE extracts obtained under optimal conditions (60% EtOH (*v*/*v*), sample/solvent ratio 1:20 (*m*/*v*)) depending on extracts pH. FJ′—frozen jostaberry; FDJ′—freeze-dried jostaberry; DJ′—oven-dried jostaberry, obtained in optimal conditions. UAE-20—jostaberry extract obtained by ultrasound-assisted extraction 20 min; MAE 100-6—jostaberry extract obtained by microwave-assisted extraction, magnetron power 100 W, 6 min; MAE 180-6—jostaberry extract obtained by microwave-assisted extraction, magnetron power 180 W, 6 min; MAE 300-6—jostaberry extract obtained by microwave-assisted extraction, magnetron power 300 W, 6 min. The results are presented as the mean of three measurements ± standard deviation (SD). Different letters (^a–l^) designate statistically different results (*p* ≤ 0.05).

The most stable intense red color is characteristic of jostaberry extracts acidulated at a pH of 2.5 (Figure 2a,b). This is due to the protonated form of the flavylium cation of anthocyanins, characteristic for an acid environment (pH < 3). At a pH between 4 and 5, anthocyanins will be in the unprotonated form, chromanol (flavonol), and their red color loses its intensity [78]. At the same time, the free radical scavenging activity of polyphenol compounds from the extracts FJ′, FDJ′, and DJ′ depend on the surrounding pH of the reaction environment. With the increase in pH from 2.5 to 3.5, at 4.5, in the TEAC assay, this increases the activity of capturing the DPPH^•^ radical and the free-cation radical ABTS^•+^. Ghosh S. et al. mentioned that AA (determined by DPPH and the Photochem method) of palm wine and palm vinegar increase as the pH increases: 3.5 < 4.5 < 5.5 [79].

Some authors have shown that polyphenols, such as hydroxyflavones and anthocyanins, contain more OH groups, which with the increase in the pH value of the environment will have a higher dissociation rate. Also, electron transfer is pH-dependent [66]. Anthocyanidins are considered strong radical scavengers, especially in slightly alkaline conditions, followed by flavonoids and phenolic acids. Thus, with the increase in the pH value and the deprotonation of the hydroxyl groups, the antiradical activity of the polyphenols increases [80].

### 3.9. The Influence of Jostaberry Extract Storage Conditions on Color Parameters and AA

**Figure 3:**
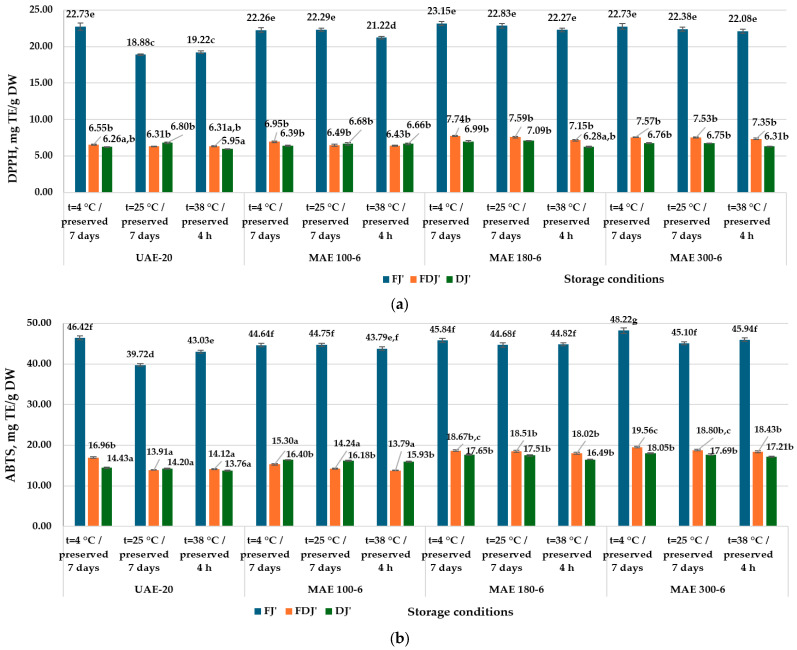
AA by DPPH (**a**) and ABTS (**b**) in UAE and MAE jostaberry extracts obtained under optimal conditions (60% EtOH (*v*/*v*), sample/solvent ratio 1:20 (*m*/*v*)) depending on the storage conditions of the extracts. FJ′—frozen jostaberry; FDJ′—freeze-dried jostaberry; DJ′—oven dried jostaberry, obtained in optimal conditions. UAE-20—jostaberry extract obtained by ultrasound-assisted extraction 20 min; MAE 100-6—jostaberry extract obtained by microwave-assisted extraction, magnetron power 100 W, 6 min; MAE 180-6—jostaberry extract obtained by microwave-assisted extraction, magnetron power 180 W, 6 min; MAE 300-6—jostaberry extract obtained by microwave-assisted extraction, magnetron power 300 W, 6 min. The results are presented as the mean of three measurements ± standard deviation (SD). Different letters (^a–g^) designate statistically different results (*p* ≤ 0.05).

The extracts of FJ′, FDJ′, and DJ′, obtained under optimal conditions, were stored under different conditions (temperature and time, specific to the confectionery product and fermented dairy product technologies) and used for the determination the color parameters (Table S4) and TEAC by DPPH and ABTS (Figure 3a,b).

The research carried out confirmed again that the drying process of the berries most influenced the color parameters and the AA of jostaberry UAE and MAE extracts. The AA of jostaberry extracts was less influenced by the variation in temperature and storage time.

The DPPH and ABTS inhibition capacity in FJ′ extracts stored at 25 °C for 7 days and in extracts stored at 38 °C for 4 h decreased in the medium by 4.39 and 6.69%, and by 5.88 and 4.06%, respectively, compared to FJ′ extracts stored at 4°C for 7 days.

In the case of FDJ′ and DJ′ extracts, storage temperature and time did not affect AA. It is possible that the ethyl alcohol contained in jostaberry extracts preserved the antioxidant properties of the BAC under the indicated conditions.

The bibliographic data attest that the DPPH scavenging ability of *Momordica Charantia* L. juices with the addition of benzoic acid, after 3 days of storage, decreased by 97.41% in the samples kept at 37 °C, by 54.36% in the samples kept at 25 °C, and by 5.15% in samples stored at 4 °C [81].

Likewise, the color parameters of the josta extracts did not undergo essential changes under the predetermined storage conditions (Table S4). The values for the L* of jostaberry UAE and MAE extracts do not undergo major changes depending on the method of pretreatment of jostaberries. Maximum values were recorded for FJ′ and DJ′. The lowest L* was obtained for MAE extracts obtained from freeze-dried jostaberries.

Keeping the samples at temperatures of 4 °C led to the greatest decrease in the brightness of the extracts, followed by the treatment at 38 °C and the maximum L* was for the jostaberry extracts kept at 25 °C.

The a* presented positive values for all the analyzed extracts. The obtained values showed a more intense red color in the FJ′ and FDJ′. For the DJ′, the a* values were the lowest, stored at 4 °C and 25°C. All the josta extracts kept at the temperature of 38 °C showed practically the lowest intensity of the red color.

The b* values were obviously higher for the DJ′ compared to FJ′ and FDJ′, which indicates a greater yellow color intensity.

Pateiro et al. reported that air-drying processing of jostaberries at 103 °C, for 4 h resulted in crust formation, lack of flavor, and browning reactions (enzymatic and non-enzymatic) [82].

The most insignificant changes in color (12.25%) and shrinkage (17.21%) were obtained in the fruits subjected to the freeze-drying process, and the highest values for these parameters (45.57% and 66.75%, respectively) were recorded for fruits treated by drying with hot air [83,84].

### 3.10. Mathematical Modeling

The mutual information analysis was applied to determine the influence of ultrasound (5, 10, 15, 20, 25, and 30 min) and microwave (2, 4, and 6 min) durations at different magnetron powers (100, 180, and 300 W) on TPC, TFC, TA, AA (DPPH, ABTS), pH, and color parameters (L*, a*, b*, C* and h*) in all jostaberry extracts (FJ, FDJ, DJ), Table 6.

The data in Table 6 show that the duration of ultrasound application significantly influenced TA content (mutual information 0.367 bits) and TFC (mg QE/g DW) (0.329 bits). Increasing the duration of ultrasound application influenced TPC (0.212 bits), TFC (mg RuE/g DW) (0.199 bits), and AA: ABTS (0.124 bits) and DPPH (0.104 bits). The pH of the jostaberry extracts was slightly influenced, with the mutual information value being 0.048 bits. In the case of color parameters, the duration of ultrasound application influenced L* and h* to an equal extent, with the mutual information value being 0.141 bits. Other color parameters such as a*, b*, and C* were insignificantly influenced, the values being 0.010, 0.020, and 0.001 bits, respectively.

In the MAE, an application time of 2, 4, and 6 min, at all magnetron powers (100, 130, and 300 W) significantly influenced TA content (0.333 bits), followed by TFC (mg QE/g DW) (0.315 bits), AA: ABTS (0.259 bits), and DPPH (0.241 bits). In the case of color parameters, microwaves influenced the most b* (0.278 bits) and a* (0.222 bits). Other color parameters are presented in the following descending order: C* (0.167 bits), L* (0.130 bits), and h* (0.129 bits). The pH of the jostaberry extracts obtained in the MAE was influenced more than in the case of UAE, the value being 0.111 bits, Table 6.

Table S5 shows the influence of the extraction medium pH, treatment temperature, and storage time on AA (DPPH, ABTS) and color parameters (L*, a*, b*, C*, and h*) in the jostaberry extracts (FJ′, FDJ′, DJ′) obtained under optimal extraction conditions with the application of ultrasound (UAE-20) and microwaves (MAE 100-6; MAE 180-6; MAE 300-6).

In the case of the pH in jostaberry extracts, it was found that it has an important influence in the application of UAE. Thus, the effect of the pH changes greatly influenced the AA: ABTS (0.499 bits) and DPPH (0.389 bits). The color parameters, especially the C*, was influenced more than the L*, the values being 0.333 bits and 0.055 bits, respectively. The h* and the b* were influenced by pH equally, the value being 0.278 bits, Table S5.

In the MAE jostaberry extracts, the pH also strongly influenced the AA: DPPH (0.426 bits) and ABTS (0.315 bits). L* and a* were equally influenced, the mutual information value being 0.241 bits, which were followed in descending order by: b* (0.203 bits), C* (0.204 bits), and h* (0.130 bits), Table S5.

The results of the mutual information in Table S5 show that the thermal treatment and storage time of jostaberry MAE extracts influenced more the DPPH AA (0.352 bits), followed by C* (0.278 bits) and a* (0.259 bits). L* and h* were equally influenced, the value being 0.111 bits. In the case of UAE jostaberry extracts, the important effect of thermal treatment and storage time was on ABTS AA (0.278 bits). C* and a* were equally influenced, the mutual information value was 0.222 bits. The smallest influence was on L* (0.055 bits), Table S5.

Mutual information analysis was applied to determine the influence of pH values on pectin properties from apple pomace obtained by UAE and MAE [49]; and on the AA and CIELab parameters of rosehip extracts [85]. Also, the same model was used to investigate the effect of extraction temperature on the content of bioactive compounds in grape marc extracts [86].

## 4. Conclusions

Phytochemical, antimicrobial, and antioxidant activity, as well as the color parameters, of UAE and MAE extracts from frozen, freeze-dried, and oven-dried jostaberries grown in Moldova were analyzed. The optimal extraction conditions were selected after determination of TPC, TFC, TA, DPPH, and ABTS AA in FJ, FDJ, and DJ extracts.

Research has shown that non-conventional extraction methods are less destructive to anthocyanins; meanwhile, drying the berries reduced TA, regardless of the extraction method. The oven-drying process reduced the concentration of TA by 99.4% and of ascorbic acid by 92.42% compared to FJ. The chlorogenic acid concentration decreased in the UAE and MAE extracts of FDJ by about 31.9% and 25.3%, and in DJ extracts by 66.5% and 60.4%, respectively, compared to FJ.

The freeze-drying and oven-drying procedures contributed to the reduction in the AA of jostaberry extracts. In all extraction conditions, TEAC test by ABTS recorded AA values (mg TE/g DW) between 35.64 and 109.17 for FJ extracts, 45.73 and 82.22 for FDJ and 34.04 and 52.20 for DJ extracts. AA by the DPPH method varied between 17.70 and 35.26; 7.50 and 7.96, and 6.31 and 7.40 mg TE/g DW of FJ, FDJ, and DJ extracts, respectively. Jostaberry pretreatment produced significant changes in all color parameters. The red tone predominates in the FJ and FDJ extracts, and the orange tone in the DJ ones. The most stable intense red color is characteristic of jostaberry extracts acidulated at pH 2.5, due to the protonated form of the flavylium cation of anthocyanins.

Mutual information analysis was applied to determine the influence of ultrasound and microwave durations, different magnetron power on TPC, TFC, TA, AA (DPPH, ABTS), pH, and color parameters in FJ, FDJ, and DJ. TA (0.333 bits) and TFC (0.329 bits) were most significantly influenced. The DPPH and ABTS inhibition capacity of all FJ′ extracts has higher values and varies more strongly, depending on pH, heat treatment and storage time, compared to the AA values of FDJ′ and DJ′ extracts. The antimicrobial activity of FJP, FDJP and DJP on Gram-positive bacteria (*Bacillus cereus*, *Staphylococcus aureus*), Gram-negative bacteria (*Escherichia coli*, *Pseudomonas aeruginosa*, *Salmonella* Abony), and fungi (*Candida albicans*) was tested in vitro. A significant antimicrobial effect was observed on all bacterial strains studied for FJP. FDJP was more active on *Bacillus cereus*, *Staphylococcus aureus*, and *Escherichia coli*. DJP was more active on *Salmonella* Abony and *Pseudomonas aeruginosa*. The antifungal effect of DJP was stronger compared to FDJP.

The results obtained in this study demonstrate that the processing technologies of vegetable raw materials must focus on the implementation of harmless methods that do not allow oxidative decomposition, either by enzymatic means or by thermal degradation of the polyphenols and vitamins responsible for the beneficial effects on health consumers.

## Figures and Tables

**Table 1:** Chromatographical and statistical parameters of HPLC-PDA detection of compounds identified in jostaberry extracts.

Used Standard Compound	PDA Maximum, nm	Retention Time, min	Detection Limit, mg/L	R^2^	Linear Range, mol/L
Ascorbic Acid	244	4.3	0.27	0.99	(1.10–11.0) × 10^−5^
Cyanidine-3-O-Glucoside	520	12.3	0.29	0.97	(0.41–4.10) × 10^−5^
Chlorogenic Acid	325	13.5	0.30	0.98	(0.56–5.60) × 10^−5^
Caffeic Acid	325	14.0	0.23	0.99	(1.10–11.0) × 10^−5^
Rutin (Quercetin-3-O-rutoside)	355	17.6	0.68	0.98	(0.35–3.50) × 10^−5^

HPLC-PDA: high-performance liquid chromatography with photo diode array; R^2^—regression coefficient.

**Table 2:** Chemical composition of jostaberries.

Indices	Jostaberries		
	FJ	FDJ	DJ
Dry matter, %	19.76 ± 0.45 ^a^	92.00 ± 1.02 ^b^	96.50 ± 0.98 ^b,c^
Protein content, %	1.00 ± 0.02 ^a^	3.75 ± 0.15 ^b^	3.65 ± 0.08 ^b^
Fat content, %	0.70 ± 0.04 ^a^	2.05 ± 0.06 ^c^	1.70 ± 0.07 ^b^
Ash content, %	0.82 ± 0.02 ^a^	3.86 ± 0.05 ^b^	3.96 ± 0.04 ^c^

FJ—frozen jostaberries; FDJ—freeze-dried jostaberries; DJ—oven-dried jostaberries. The results are presented as the mean of three measurements ± standard deviation (SD). Different letters (^a–c^) designate statistically different results (*p* ≤ 0.05).

**Table 3:** Antimicrobial activity of the jostaberry preparations against bacteria and yeast strains.

Test Strains	Zone of Inhibition, mm *			MIC, mg/mL			MBC/MFC, mg/mL			MIC, mg/mL	DMSO
	FJP	FDJP	DJP	FJP	FDJP	DJP	FJP	FDJP	DJP	Tetracycline	
Gram-positive bacteria											
*Staphylococcus aureus*	18.0 ± 0.7 ^c,d^	23.0 ± 0.6 ^d,e^	18.0 ± 0.5 ^c,d^	10.6 ± 0.2 ^b^	13.7 ± 0.8 ^b,c^	20.8 ± 0.5 ^d^	10.6 ± 0.1 ^b^	27.5 ± 0.5 ^e^	41.6 ± 1.3 ^f^	1.0 ± 0.1 ^a^	N/E
*Bacillus cereus*	23.0 ± 1.3 ^e,f^	26.0 ± 0.0 ^f^	25.0 ± 0.8 ^f^	2.7 ± 0.1 ^b^	3.4 ± 0.2 ^b^	5.2 ± 0.4 ^b,c^	2.7 ± 0.2 ^b^	6.8 ± 0.7 ^c^	10.4 ± 0.4 ^d^	1.2 ± 0.1 ^a^	N/E
Gram-negative bacteria											
*Escherichia coli*	13.0 ± 0.8 ^b^	23.0 ± 1.0 ^d^	18.0 ± 0.7 ^c^	10.6 ± 0.5 ^b^	13.7 ± 0.7 ^b^	20.8 ± 1.2 ^c,d^	10.6 ± 0.6 ^b^	27.5 ± 1.3 ^e^	41.6 ± 1.3 ^f^	5.0 ± 0.0 ^a^	N/E
*Salmonella* Abony	13.0 ± 0.6 ^b^	10.0 ± 0.5 ^b^	10.0 ± 0.3 ^b^	10.6 ± 0.7 ^b^	55.0 ± 1.6 ^e^	41.6 ± 1.3 ^d^	21.2 ± 0.9 ^c^	110.0 ± 0.0 ^g^	83.0 ± 1.0 ^f^	5.0 ± 0.1 ^a,b^	N/E
*Pseudomonas aeruginosa*	13.0 ± 0.5 ^b,c^	17.0 ± 0.8 ^d,e^	18.0 ± 0.7 ^e^	10.6 ± 0.3 ^a^	13.7 ± 0.6 ^c^	10.4 ± 0.2 ^a^	10.6 ± 0.3 ^a^	27.5 ± 0.5 ^g^	20.8 ± 0.2 ^f^	12.5 ± 0.5 ^b^	N/E
Yeast										Miconazole	
*Candida albicans*	N/E	16.0 ± 0.3 ^a^	15.0 ± 0.5 ^a^	N/E	110.0 ± 1.1 ^c^	83.0 ± 0.9 ^b^	N/E	220.0 ± 2.0 ^e^	166.0 ± 1.8 ^d^	16.0 ± 0.3 ^a^	N/E

* Diameter of inhibition zone. FJP—frozen jostaberry preparation; FDJP—freeze-dried jostaberry preparation; DJP—oven-dried jostaberry preparation; MIC—minimum inhibitory concentration; MBC—minimum bactericidal concentration; MFC—minimum fungicidal concentration; N/E—no effect. The results are presented as the mean of three measurements ± standard deviation (SD). Different letters (^a–g^) designate statistically different results (*p* ≤ 0.05).

**Table 4:** Compounds identified and quantified by the HPLC and capillary electrophoresis methods in jostaberry extracts (sample/solvent ratio 1:100 (*m*/*v*)).

Jostaberry Extracts		Anthocyanins after Cy-3-O-Glucoside, mg/g DW	Ascorbic Acid, mg/g DW	Chlorogenic Acid, mg/g DW	Caffeic Acid, mg/g DW	Rutoside, mg/g DW	Citric Acid, mg/g DW	Malic Acid, mg/g DW
UAE-20	FJ	17.15 ± 0.03 ^f^	2.00 ± 0.17 ^c^	2.60 ± 0.19 ^f,g^	0.242 ± 0.005 ^g^	1.28 ± 0.05 ^f^	0.82 ± 0.02 ^b^	7.83 ± 0.05 ^d^
	FDJ	7.48 ± 0.08 ^d^	1.43 ± 0.02 ^b^	1.77 ± 0.04 ^d^	0.162 ± 0.001 ^c^	0.73 ± 0.03 ^d^	1.61 ± 0.05 ^d^	2.02 ± 0.01 ^b^
	DJ	0.11 ± 0.02 ^a^	0.55 ± 0.03 ^a^	0.87 ± 0.03 ^b^	0.180 ± 0.001 ^e^	0.74 ± 0.03 ^d^	1.33 ± 0.03 ^c^	10.81 ± 0.07 ^e^
MAE 100-6	FJ	2.42 ± 0.17 ^b^	3.60 ± 0.26 ^d,e^	1.80 ± 0.17 ^d,e^	0.165 ± 0.002 ^c,d^	0.30 ± 0.03 ^a^	0.91 ± 0.04 ^b^	1.16 ± 0.01 ^a^
	FDJ	6.71 ± 0.26 ^d^	4.03 ± 0.17 ^e^	1.78 ± 0.03 ^d^	0.163 ± 0.004 ^c,d^	0.72 ± 0.02 ^d^	2.04 ± 0.06 ^e^	2.34 ± 0.03 ^b^
	DJ	0.12 ± 0.02 ^a^	0.46 ± 0.03 ^a^	0.70 ± 0.02 ^a^	0.149 ± 0.003 ^b,c^	0.53 ± 0.02 ^b,c^	1.35 ± 0.02 ^c^	10.85 ± 0.04 ^e^
MAE 180-6	FJ	6.58 ± 0.42 ^c,d^	3.47 ± 0.16 ^d^	2.15 ± 0.18 ^e,f^	0.184 ± 0.002 ^e^	0.77 ± 0.04 ^d^	1.06 ± 0.02 ^b^	1.15 ± 0.01 ^a^
	FDJ	5.61 ± 0.21 ^c^	3.31 ± 0.15 ^d^	1.58 ± 0.04 ^d^	0.134 ± 0.003 ^a,b^	0.62 ± 0.03 ^c^	3.31 ± 0.03 ^f^	4.12 ± 0.02 ^c^
	DJ	0.09 ± 0.02 ^a^	0.42 ± 0.03 ^a^	0.74 ± 0.03 ^a,b^	0.168 ± 0.004 ^d^	0.55 ± 0.02 ^b,c^	1.44 ± 0.01 ^c^	11.61 ± 0.09 ^f^
MAE 300-6	FJ	7.90 ± 0.50 ^d,e^	7.52 ± 0.48 ^g^	3.28 ± 0.01 ^h^	0.271 ± 0.004 ^h^	1.05 ± 0.04 ^e^	0.72 ± 0.02 ^a,b^	0.92 ± 0.01 ^a^
	FDJ	8.59 ± 0.30 ^e^	6.79 ± 0.21 ^f^	2.45 ± 0.05 ^f^	0.217 ± 0.003 ^f^	0.98 ± 0.02 ^e^	3.63 ± 0.0 ^f^	4.54 ± 0.07 ^c^
	DJ	0.09 ± 0.02 ^a^	0.57 ± 0.03 ^a^	1.30 ± 0.04 ^e^	0.216 ± 0.003 ^f^	0.96 ± 0.01 ^e^	1.41 ± 0.01 ^c^	11.93 ± 0.05 ^f^

FJ—frozen jostaberry; FDJ—freeze-dried jostaberry; DJ—oven-dried jostaberry. UAE-20—jostaberry extract obtained by ultrasound-assisted extraction 20 min; MAE 100-6—jostaberry extract obtained by microwave-assisted extraction, magnetron power 100 W, 6 min; MAE 180-6—jostaberry extract obtained by microwave-assisted extraction, magnetron power 180 W, 6 min; MAE 300-6—jostaberry extract obtained by microwave-assisted extraction, magnetron power 300 W, 6 min. The results are presented as the mean of three measurements ± standard deviation (SD). Different letters (^a–h^) designate statistically different results (*p* ≤ 0.05).

**Table 5:** Color parameters of jostaberry UAE and MAE extracts (60% EtOH (*v*/*v*), sample/solvent ratio 1:20 (*m*/*v*)).

Jostaberry Extracts		L*			a*			b*			C*			h*, °		
Extraction Methods	Time, min	FJ	FDJ	DJ	FJ′	FDJ	DJ	FJ	FDJ	DJ	FJ	FDJ	DJ	FJ	FDJ	DJ
UAE	5	67.21 ± 0.65 ^j^	54.96 ± 0.19 ^f^	57.87 ± 0.27 ^f,g^	25.04 ± 0.08 ^c^	55.03 ± 0.29 ^i^	37.83 ± 0.17 ^e,f^	2.21 ± 0.02 ^a^	1.52 ± 0.01 ^a^	50.70 ± 0.23 ^h^	25.14 ± 0.09 ^c^	55.05 ± 0.33 ^g^	63.26 ± 0.46 ^h^	5.04 ± 0.14 ^b^	1.58 ± 0.07 ^a^	53.27 ± 0.30 ^i^
	10	68.84 ± 0.71 ^j,k^	55.63 ± 0.42 ^f^	56.46 ± 0.28 ^f^	26.90 ± 0.11 ^c^	45.88 ± 0.31 ^g^	42.92 ± 0.24 ^g^	2.18 ± 0.02 ^a^	4.65 ± 0.01 ^b^	48.16 ± 0.17^g^	26.99 ± 0.07 ^c^	46.12 ± 0.26 ^f^	64.51 ± 0.40 ^h,i^	4.63 ± 0.09 ^b^	5.79 ± 0.03 ^b^	48.29 ± 0.27 ^h^
	15	70.22 ± 0.44 ^k^	56.22 ± 0.38 ^f^	53.58 ± 0.31 ^e^	25.15 ± 0.16 ^c^	40.86 ± 0.24 ^f^	46.98 ± 0.32 ^g^	1.96 ± 0.03 ^a^	7.04 ± 0.06 ^b^	44.16 ± 0.14 ^f^	25.23 ± 0.13 ^c^	41.46 ± 0.31 ^e,f^	64.48 ± 0.36 ^h,i^	4.46 ± 0.07 ^b^	9.78 ± 0.15 ^c^	43.23 ± 0.24 ^g^
	20	69.22 ± 0.61 ^j,k^	56.80 ± 0.41 ^f^	51.41 ± 0.38 ^d,e^	27.51 ± 0.19 ^c^	57.52 ± 0.02^j^	49.92 ± 0.24 ^h^	2.09 ± 0.02 ^a^	2.10 ± 0.01 ^a^	41.33 ± 0.18 ^f^	27.59 ± 0.10 ^c^	57.56 ± 0.37 ^g^	64.81 ± 0.33 ^h,i^	4.34 ± 0.05 ^b^	2.09 ± 0.08 ^a^	39.62 ± 0.31 ^f^
	25	77.28 ± 0.54 ^l^	58.65 ± 0.38 ^f^	53.72 ± 0.30 ^e^	21.89 ± 0.15 ^b^	43.50 ± 0.29 ^g^	45.06 ± 0.33 ^g^	2.18 ± 0.01 ^a^	9.11 ± 0.02 ^c^	57.64 ± 0.11 ^i^	22.00 ± 0.04 ^b^	44.44 ± 0.21 ^f^	73.16 ± 0.26 ^j^	5.69 ± 0.08 ^b^	11.83 ± 0.04 ^c^	51.98 ± 0.19 ^i^
	30	79.64 ± 0.39 ^m^	62.00 ± 0.29 ^h^	53.56 ± 0.38 ^e^	16.5 ± 0.26 ^a^	41.11 ± 0.36 ^f^	42.30 ± 0.31 ^f,g^	2.57 ± 0.02 ^a^	12.55 ± 0.05 ^d^	63.64 ± 0.22 ^j^	16.70 ± 0.27 ^a^	42.98 ± 0.15 ^f^	76.42 ± 0.31 ^j,k^	8.85 ± 0.04 ^c^	16.98 ± 0.08 ^e^	56.39 ± 0.31 ^j^
MAE, 100 W	2	67.95 ± 0.42 ^j^	60.08 ± 0.36 ^g^	64.05 ± 0.29 ^i^	32.84 ± 0.30 ^c^	57.03 ± 0.43 ^j^	39.85 ± 0.36 ^f^	2.73 ± 0.03 ^a,b^	14.48 ± 0.06 ^d^	67.21 ± 0.18 ^k^	32.95 ± 0.19 ^d^	58.84 ± 0.42 ^g,h^	78.14 ± 0.27 ^k^	4.75 ± 0.03 ^b^	14.25 ± 0.26 ^d^	59.34 ± 0.28 ^j,k^
	4	65.65 ± 0.58 ^i^	48.07 ± 0.27 ^c^	57.52 ± 0.48 ^f,g^	39.64 ± 0.25 ^f^	60.51 ± 0.38 ^j^	42.77 ± 0.26 ^g^	2.42 ± 0.02 ^a^	13.46 ± 0.08 ^d^	57.55 ± 0.11 ^i^	39.71 ± 0.32 ^e^	61.99 ± 0.32 ^h^	71.70 ± 0.35 ^j^	3.49 ± 0.02 ^b^	12.54 ± 0.13 ^d^	53.38 ± 0.25 ^i^
	6	64.32 ± 0.49 ^i^	46.44 ± 0.45 ^c^	53.14 ± 0.31 ^e^	41.11 ± 0.20 ^f^	67.02 ± 0.31 ^l^	49.13 ± 0.27 ^h^	1.88 ± 0.01 ^b^	11.94 ± 0.02 ^c^	52.16 ± 0.33 ^h^	41.15 ± 0.26 ^e,f^	68.08 ± 0.20 ^i^	71.65 ± 0.27 ^j^	2.63 ± 0.05 ^a^	10.10 ± 0.04 ^c^	46.71 ± 0.38 ^h^
MAE, 180 W	2	69.14 ± 0.31 ^j^	67.40 ± 0.35 ^j^	56.10 ± 0.28 ^f^	27.59 ± 0.21 ^c^	56.81 ± 0.40 ^i,j^	47.79 ± 0.38 ^h^	3.02 ± 0.01 ^b^	17.05 ± 0.14 ^e^	65.37 ± 0.28 ^k^	27.75 ± 0.18 ^c^	59.31 ± 0.30 ^h^	80.98 ± 0.40 ^k^	6.25 ± 0.03 ^b^	16.71 ± 0.09 ^d^	53.83 ± 0.25 ^i^
	4	67.03 ± 0.55 ^j^	51.22 ± 0.27 ^d^	52.55 ± 0.32 ^e^	30.15 ± 0.05 ^d^	60.65 ± 0.06 ^j^	50.75 ± 0.13 ^h^	2.87 ± 0.02 ^b^	18.63 ± 0.07 ^e^	62.74 ± 0.13 ^j^	30.29 ± 0.02 ^d^	65.36 ± 0.30 ^i^	80.70 ± 0.37 ^k^	5.44 ± 0.09 ^b^	16.56 ± 0.10 ^d^	51.03 ± 0.12 ^i^
	6	62.89 ± 0.47 ^h^	48.23 ± 0.42 ^c^	46.61 ± 0.31 ^c^	34.08 ± 0.08 ^e^	69.49 ± 0.16 ^l^	58.67 ± 0.21 ^j^	2.05 ± 0.04 ^a^	11.14 ± 0.02 ^c^	58.82 ± 0.19 ^i^	34.14 ± 0.06 ^d^	70.38 ± 0.12 ^i^	83.08 ± 0.28 ^l^	3.44 ± 0.08 ^b^	9.11 ± 0.14 ^c^	45.07 ± 0.27 ^g,h^
MAE, 300 W	2	71.45 ± 0.31 ^k^	61.43 ± 0.16 ^h^	57.82 ± 0.11 ^g^	25.58 ± 0.18 ^c^	53.06 ± 0.21 ^i^	46.46 ± 0.15 ^g^	2.59 ± 0.01 ^a^	15.12 ± 0.02 ^d^	66.66 ± 0.21 ^k^	25.71 ± 0.09 ^c^	55.17 ± 0.18 ^g^	81.25 ± 0.26 ^k^	5.78 ± 0.04 ^b^	15.91 ± 0.07 ^d^	55.12 ± 0.23 ^j^
	4	68.76 ± 0.53 ^j^	45.80 ± 0.23 ^b,c^	53.07 ± 0.18 ^e^	32.19 ± 0.11 ^d^	56.97 ± 0.15 ^j^	45.15 ± 0.21 ^g^	2.36 ± 0.05 ^a^	11.23 ± 0.09 ^d^	64.29 ± 0.11 ^k^	32.28 ± 0.21 ^d^	68.07 ± 0.34 ^i^	78.56 ± 0.39 ^k^	4.19 ± 0.06 ^b^	11.15 ± 0.09 ^c^	54.92 ± 0.11 ^j^
	6	65.03 ± 0.48 ^j^	42.56 ± 0.20 ^a^	43.11 ± 0.31 ^b^	35.16 ± 0.03 ^e^	60.18 ± 0.41 ^j^	48.80 ± 0.29 ^h^	2.01 ± 0.04 ^a^	7.06 ± 0.21 ^b^	59.10 ± 0.18 ^i,j^	35.22 ± 0.02 ^d^	60.59 ± 0.28 ^h^	76.64 ± 0.42 ^j,k^	3.27 ± 0.29 ^b^	6.69 ± 0.21 ^b^	50.45 ± 0.39 ^i^

FJ—frozen jostaberry; FDJ—freeze-dried jostaberry; DJ—oven-dried jostaberry, obtained in optimal conditions. L*—lightness; a*—red-green parameter; b*—yellow-blue parameter; C*—chromaticity; h*—hue angle. The results are presented as the mean of three measurements ± standard deviation (SD). Different letters (^a–m^) designate statistically different results (*p* ≤ 0.05).

**Table 6:** Results of mutual analysis of the influence of ultrasound (5, 10, 15, 20, 25, and 30 min) and microwave (2, 4, and 6 min) durations at different magnetron powers (100, 180, and 300 W) on the content of BAC, AA, pH, and color parameters in all jostaberry extracts (FJ, FDJ, DJ).

Parameter	Influence of Extraction Conditions, bits	
	Duration of UAE	Duration of MAE at Different Magnetron Powers
Total polyphenol content	0.212	0.204
Total flavonoid content		
mg RuE/g DW	0.199	0.240
mg QE/g DW	0.329	0.315
Anthocyanins content	0.367	0.333
Antioxidant activity (DPPH)	0.104	0.241
Antioxidant activity (ABTS)	0.124	0.259
pH	0.048	0.111
Lightness, L*	0.141	0.130
Red–green parameter, a*	0.010	0.222
Yellow–blue parameter, b*	0.020	0.278
Chromaticity, C*	0.001	0.167
Hue angle, h*	0.141	0.129

## Data Availability

The original contributions presented in the study are included in the article; further inquiries can be directed to the corresponding author.

## Supplementary Materials

The following supporting information can be downloaded at: https://www.mdpi.com/article/10.3390/antiox13080890/s1, Figure S1: Photos of the pretreated jostaberry: (a) frozen jostaberry; (b) freeze-dried jostaberry; (c) oven-dried jostaberry; Figure S2: TFC, mg QE/g DW (a,b) and AA by DPPH (c,d) in UAE and MAE extracts of FJ, FDJ, and DJ (60% EtOH (*v*/*v*), sample/solvent ratio 1:100 (*m*/*v*)); Table S1: pH values in FJ, FDJ, and DJ extracts; Table S2: Coefficients of correlation (R^2^) between AA and content of BAC determined in jostaberry extracts; Table S3: The color parameters in UAE and MAE jostaberry extracts obtained under optimal conditions (60% EtOH (*v*/*v*), sample/solvent ratio 1:20 (*m*/*v*)) depending on extracts pH; Table S4: Color parameters in UAE and MAE jostaberry extracts obtained under optimal conditions (60% EtOH (*v*/*v*), sample/solvent ratio 1:20 (*m*/*v*)) depending on the storage conditions of the extracts; Table S5: The influence of pH and storage conditions on AA (DPPH, ABTS) and color parameters (L*, a*, b*, C* and h*) of jostaberry extracts obtained under optimal conditions with the application of UAE and MAE (FJ′, FDJ′, DJ′).
