# Supplementary material for: Phytochemical, Antimicrobial, and Antioxidant Activity of Different Extracts from Frozen, Freeze-Dried, and Oven-Dried Jostaberries Grown in Moldova
Source: Antioxidants (Basel). 2024 Jul 23;13(8):890. doi: 10.3390/antiox13080890 (PMC11351914; doi:10.3390/antiox13080890)
Supplement: Supplementary file 1 [file antioxidants-13-00890-s001.zip › antioxidants-3091566-supplementary.pdf]

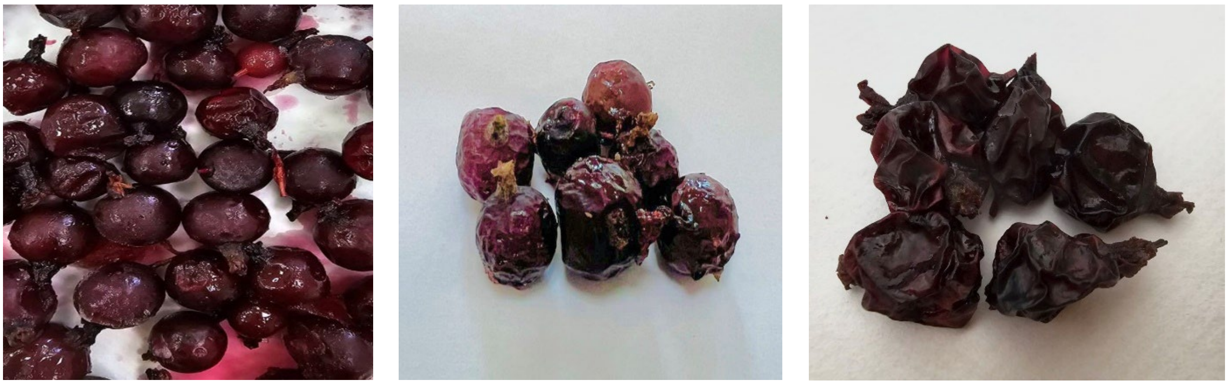

a) b) c)

Figure S1. Photos of the pretreated jostaberry: a) frozen jostaberry; b) freeze dried jostaberry; c) oven-dried jostaberry.

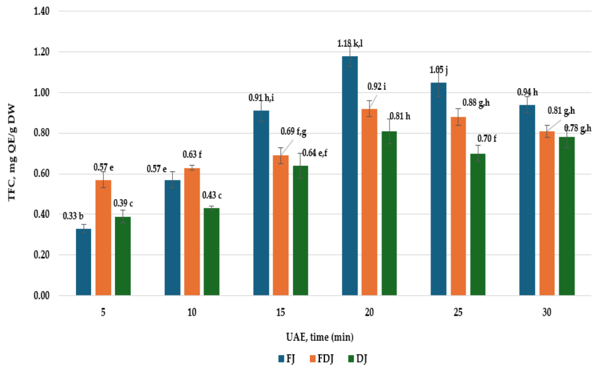

a)

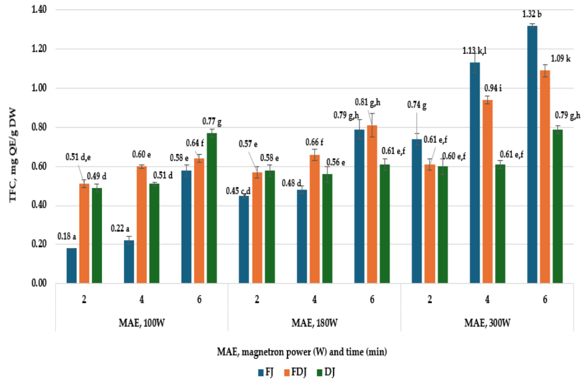

b)

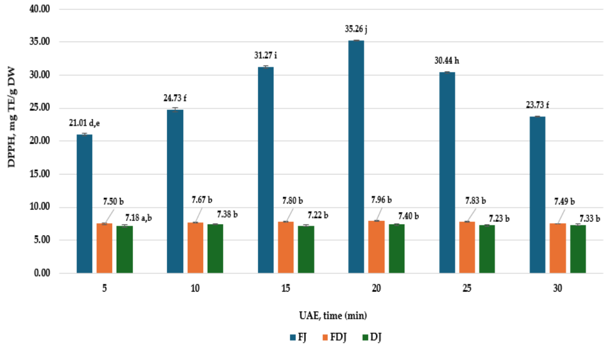

c)

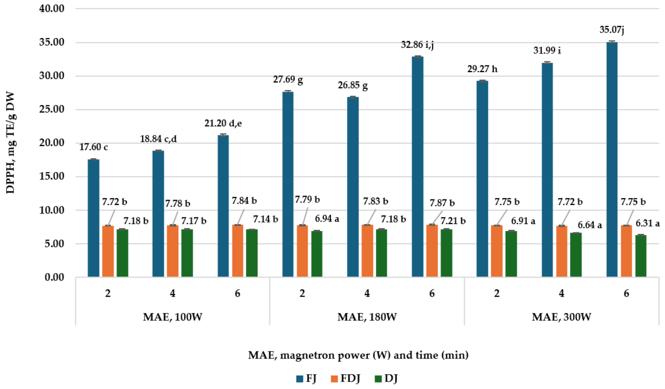

d)

Figure S2. TFC, mg QE/g DW (a, b) and AA by DPPH (c, d) in UAE and MAE extracts of FJ, FDJ and DJ (60 % EtOH (*v*/*v*), sample:solvent ratio 1:100 (*m*/*v*)).

FJ - frozen jostaberry; FDJ – freeze-dried jostaberry; DJ - oven-dried jostaberry; TPC – total polyphenol content; GAE – gallic acid equivalent; DW - dry weight; TFC - total flavonoid content; QE - quercetin equivalent; TA – total anthocyanin; Cy3GE - cyanidin-3-glucoside equivalent; AA – antioxidant activity; TE - trolox equivalent. The results are presented as the mean of three measurements ± standard deviation (SD). Different letters ($^{a–m}$) designate statistically different results ($p \leq 0.05$).

Table S1. pH values in FJ, FDJ, DJ extracts

Extraction methods	Time, min	Extracts pH values		
		Jostaberry extract		
		FJ	FDJ	DJ
UAE	5	4.16±0.01 ^{e}	4.10±0.01 ^{c}	4.35±0.02 ^{k,l}
	10	4.04±0.02 ^{a,b}	4.24±0.01 ^{g,h}	4.41±0.01 ^{m,n}
	15	4.06±0.01 ^{a,b}	4.20±0.02 ^{f,g}	4.28±0.01 ^{i}
	20	4.09±0.01 ^{b,c}	4.25±0.01 ^{h}	4.31±0.01 ^{j}
	25	4.21±0.01 ^{f,g}	4.14±0.01 ^{d,e}	4.29±0.01 ^{i,j}

	30	4.13±0.01 ^{d}	4.28±0.01 ^{i}	4.23±0.01 ^{g,h}
MAE, 100W	2	4.17±0.01 ^{e,f}	4.33±0.01 ^{j,k}	4.27±0.01 ^{h,i}
	4	4.20±0.02 ^{f,g}	4.15±0.01 ^{d,e}	4.29±0.01 ^{i,j}
	6	4.19±0.01 ^{f}	4.40±0.01 ^{m}	4.26±0.01 ^{h,i}
MAE, 180W	2	4.23±0.01 ^{g,h}	4.36±0.01 ^{k,l}	4.43±0.01 ^{n}
	4	4.23±0.02 ^{g,h}	4.26±0.01 ^{h,i}	4.28±0.01 ^{i}
	6	4.26±0.01 ^{h,i}	4.34±0.01 ^{k}	4.35±0.01 ^{k,l}
MAE, 300W	2	4.27±0.01 ^{h,i}	4.33±0.01 ^{j,k}	4.35±0.01 ^{k,l}
	4	4.21±0.01 ^{f,g}	4.37±0.01 ^{l}	4.36±0.01 ^{k,l}
	6	4.16±0.01 ^{e}	4.30±0.01 ^{i,j}	4.23±0.01 ^{g,h}

FJ - frozen jostaberry; FDJ – freeze-dried jostaberry; DJ - oven-dried jostaberry. The results are presented as the mean of three measurements ± standard deviation (SD). Different letters ($^{a–n}$) designate statistically different results (p ≤ 0.05)

Table S2. Coefficients of correlation (R^2) between AA and content of BAC determined in jostaberry extracts

Total content of BAC, relative to DW	Antioxidant activity of jostaberry extract					
	DPPH, mg TE/g DW			ABTS, mg TE/g DW		
	FJ	FDJ	DJ	FJ	FDJ	DJ
	R^2					
TPC, mg GAE/ g DW	0.8390	0.4202	0.2437	0.6955	0.9028	0.5168
TFC, mg RuE/ g DW	0.7430	0.9882	0.2324	0.4845	0.7075	0.0729
TFC, mg QE/ g DW	0.7664	0.2646	0.0395	0.5630	0.6287	0.1525
TA, mg CY3GE/ g DW	0.8041	0.4944	0.4731	0.6749	0.7604	0.0654
Ascorbic acid, mg/ g DW	0.3357	0.7525	0.2425	0.1259	0.9156	0.3311
Chlorogenic acid, mg/g DW	0.7263	0.4768	0.2425	0.7621	0.9930	0.3577

FJ - frozen jostaberry; FDJ – freeze-dried jostaberry; DJ - oven-dried jostaberry. The correlation between the total content of polyphenols (TPC), flavonoids (TFC), anthocyanins (TA) and DPPH and ABTS antioxidant activity (AA) was determined according to the values recorded by the Uv-Vis spectrophotometric method; values for ascorbic and chlorogenic acids were determined by the HPLC method.

Table S3. The color parameters in UAE and MAE jostaberry extracts obtained under optimal conditions (60% EtOH (*v/v*), sample:solvent ratio 1:20 (*m/v*)) depending on extracts pH.

**Jostaberry extracts**		**Color parameter**														
		**L***			**a***			**b***			**C***			**h*, °**		
**Extraction methods**	**pH**	**FJ'**	**FDJ'**	**DJ'**	**FJ'**	**FDJ'**	**DJ'**	**FJ'**	**FDJ'**	**DJ'**	**FJ'**	**FDJ'**	**DJ'**	**FJ'**	**FDJ'**	**DJ'**
**UAE-20**	**2.5**	48.61±0.18 ^{e}	33.31±0.15 ^{a}	52.93±0.11 ^{f,g}	62.95±0.05 ^{k}	65.59±0.09 ^{l}	31.92±0.04 ^{f}	17.10±0.09 ^{h}	14.36±0.15 ^{g}	29.47±0.08 ^{m}	65.23±0.15 ^{k}	67.14±0.11 ^{k}	43.44±0.09 ^{f}	15.20±0.12 ^{d}	12.35±0.14 ^{c}	42.71±0.11 ^{g}
	**3.5**	69.57±0.15 ^{l}	66.73±0.06 ^{k}	68.38±0.11 ^{k}	26.31±0.10 ^{e}	33.51±0.07 ^{f}	14.59±0.04 ^{b}	7.19±0.07 ^{d}	3.67±0.09 ^{b}	27.42±0.06 ^{l}	27.27±0.11 ^{c}	33.71±0.08 ^{d}	31.06±0.09 ^{d}	15.28±0.13 ^{d}	6.25±0.07 ^{b}	61.98±0.12 ^{j}
	**4.5**	67.37±0.12 ^{k}	60.73±0.09 ^{i}	71.38±0.14 ^{l}	20.00±0.13 ^{c,d}	38.26±0.08 ^{g}	9.88±0.06 ^{a}	4.33±0.04 ^{c}	4.52±0.10 ^{c}	29.26±0.07 ^{m}	20.46±0.08 ^{b}	38.53±0.08 ^{e}	30.88±0.11 ^{d}	12.22±0.10 ^{c}	6.74±0.09 ^{b}	71.34±0.12 ^{k}
**MAE 100-6**	**2.5**	50.93±0.08 ^{f}	39.41±0.05 ^{b}	58.74±0.09 ^{h}	61.78±0.10 ^{k}	65.21±0.13 ^{l}	17.80±0.09 ^{c}	26.25±0.13 ^{k}	22.86±0.07 ^{j}	25.24±0.08 ^{k}	67.13±0.11 ^{k}	69.10±0.09 ^{k}	30.89±0.09 ^{d}	23.02±0.12 ^{e,f}	19.32±0.11 ^{e}	54.81±0.10 ^{i}
	**3.5**	65.67±0.03 ^{k}	61.97±0.06 ^{i}	69.23±0.07 ^{l}	43.89±0.12 ^{h}	47.16±0.07 ^{i}	15.19±0.04 ^{c}	2.90±0.10 ^{b}	1.41±0.05 ^{a}	24.23±0.09 ^{k}	43.99±0.09 ^{f}	47.18±0.07 ^{g}	28.60±0.08 ^{c}	3.78±0.08 ^{b}	1.71±0.06 ^{a}	57.92±0.10 ^{i,j}
	**4.5**	71.31±0.05 ^{l}	68.41±0.07 ^{k}	72.86±0.14 ^{m}	16.19±0.05 ^{c}	21.09±0.06 ^{d}	9.89±0.08 ^{a}	6.20±0.03 ^{c}	2.94±0.05 ^{b}	28.62±0.11 ^{l}	17.34±0.04 ^{a}	21.29±0.07 ^{b}	30.28±0.12 ^{d}	20.95±0.05 ^{e}	7.94±0.08 ^{b,c}	70.94±0.11 ^{k}
**MAE 180-6**	**2.5**	46.46±0.08 ^{d}	42.99±0.06 ^{c}	56.28±0.05 ^{h}	68.11±0.13 ^{l}	61.65±0.11 ^{l}	18.83±0.07 ^{c}	26.32±0.09 ^{k}	13.32±0.09 ^{f}	31.45±0.07 ^{m,n}	73.02±0.10 ^{l}	63.07±0.09 ^{j}	36.66±0.07 ^{e}	21.13±0.12 ^{e}	12.19±0.10 ^{c}	59.09±0.09 ^{j}
	**3.5**	66.60±0.11 ^{k}	54.98±0.09 ^{g}	64.94±0.13 ^{j}	53.07±0.17 ^{j}	73.89±0.15 ^{m}	22.61±0.05 ^{d}	20.39±0.09 ^{i}	14.72±0.13 ^{g}	32.79±0.11 ^{n}	56.85±0.13 ^{i}	75.31±0.12 ^{m}	39.83±0.09 ^{e}	21.02±0.09 ^{e}	11.27±0.11 ^{c}	55.41±0.10 ^{i}
	**4.5**	59.73±0.08 ^{i}	56.58±0.06 ^{h}	70.93±0.03 ^{l}	23.65±0.18 ^{d}	42.93±0.14 ^{h}	11.04±0.06 ^{b}	6.26±0.11 ^{c}	13.09±0.07 ^{f}	30.30±0.05 ^{m}	25.40±0.15 ^{c}	44.88±0.10 ^{f}	32.25±0.05 ^{d}	21.38±0.13 ^{e}	16.96±0.09 ^{d}	69.98±0.06 ^{k}
**MAE 300-6**	**2.5**	50.44±0.09 ^{f}	46.93±0.06 ^{e}	59.29±0.11 ^{i}	65.38±0.12 ^{l}	51.40±0.09 ^{j}	25.94±0.14 ^{e}	27.78±0.10 ^{l}	16.86±0.07 ^{h}	29.09±0.13 ^{l,m}	71.04±0.11 ^{l}	54.09±0.08 ^{h}	38.98±0.13 ^{e,f}	23.02±0.13 ^{e,f}	18.16±0.10 ^{g}	48.28±0.15 ^{h}
	**3.5**	64.49±0.06 ^{j}	51.62±0.08 ^{f}	68.83±0.05 ^{l}	53.59±0.10 ^{j}	71.65±0.16 ^{m}	21.88±0.08 ^{d}	8.32±0.06 ^{d}	18.32±0.14 ^{h}	21.87±0.09 ^{j}	28.82±0.07 ^{c}	56.82±0.12 ^{i}	74.91±0.08 ^{m}	18.81±0.13 ^{e}	16.97±0.10 ^{d}	52.79±0.35 ^{h,i}
	**4.5**	59.22±0.15 ^{h,i}	42.75±0.09 ^{c}	67.23±0.12 ^{k}	23.87±0.04 ^{d}	63.94±0.13 ^{k}	10.02±0.06 ^{a,b}	7.83±0.11 ^{d}	18.72±0.15 ^{h}	26.22±0.02 ^{k}	25.12±0.08 ^{c}	66.62±0.11 ^{k}	28.07±0.05 ^{c}	18.16±0.10 ^{e}	16.32±0.12 ^{d}	69.09±0.06 ^{k}

FJ' - frozen jostaberry; FDJ' – freeze-dried jostaberry; DJ' – oven-dried jostaberry, obtained in optimal condition. L* - lightness; a* - red-green parameter; b* - yellow-blue parameter; C* - chromaticity; h* - hue angle. UAE 20 - jostaberry extract obtained by ultrasound assisted extraction 20 minutes; MAE 100-6 – jostaberry extract obtained by microwave assisted extraction, magnetron power 100 W, 6 minutes; MAE 180-6 – jostaberry extract obtained by microwave assisted extraction, magnetron power 180 W, 6 minutes; MAE 300-6 – jostaberry extract obtained by microwave assisted extraction, magnetron power 300 W, 6 minutes. The results are presented as the mean of three measurements ± standard deviation (SD). Different letters ($^{a–n}$) designate statistically different results ($p \leq 0.05$).

Table S4. Color parameters in UAE and MAE jostaberry extracts obtained under optimal conditions (60% EtOH (*v/v*), sample:solvent ratio 1:20 (*m/v*)) depending on the storage conditions of the extracts

**Extraction methods**	**Treated jostaberry extract**	**Color parameter**														
		**L***			**a***			**b***			**C***			**h*, °**		
		**FJ'**	**FDJ'**	**DJ'**	**FJ'**	**FDJ'**	**DJ'**	**FJ'**	**FDJ'**	**DJ'**	**FJ'**	**FDJ'**	**DJ'**	**FJ'**	**FDJ'**	**DJ'**
**UAE-20**	**t=4 °C / preserved 7 days**	69.08± 0.17 ^{h}	67.41± 0.19 ^{g}	69.00± 0.14 ^{h}	20.79± 0.09 ^{f}	22.62± 0.11 ^{f}	11.30± 0.07 ^{c}	4.10± 0.03 ^{b}	7.26± 0.09 ^{c}	23.80± 0.11 ^{h}	21.19± 0.15 ^{f}	23.76± 0.17 ^{g}	26.35± 0.12 ^{g}	11.16± 0.16 ^{b}	17.79± 0.11 ^{c}	64.60± 0.09 ^{h}
	**t=25 °C / preserved 7 days**	72.89± 0.19 ^{j}	71.21± 0.20 ^{i}	75.85± 0.23 ^{k}	10.71± 0.05 ^{c}	15.82± 0.13 ^{d}	4.83± 0.06 ^{a}	4.55± 0.03 ^{b}	6.04± 0.08 ^{c}	15.35± 13 ^{e}	11.64± 0.11 ^{c}	16.93± 0.15 ^{e}	16.09± 0.18 ^{d}	23.02± 0.10 ^{d}	20.90± 0.13 ^{c}	72.53± 0.18 ^{i}
	**t=38 °C / preserved 4 h**	72.46± 0.08 ^{j}	72.19± 0.16 ^{j}	69.92± 0.15 ^{h,i}	9.03± 0.07 ^{b}	4.66± 0.02 ^{a}	8.01± 0.12 ^{b}	3.32± 0.05 ^{b}	2.82± 0.03 ^{b}	15.63± 0.19 ^{e}	9.62± 0.06 ^{b}	5.45± 0.11 ^{a}	17.56± 0.14 ^{d}	20.19± 0.09 ^{c}	31.18± 0.15 ^{e}	62.87± 0.17 ^{g}
**MAE 100-6**	**t=4 °C / preserved 7 days**	70.06± 0.22 ^{h}	64.07± 0.17 ^{d,e}	65.88± 0.27 ^{f}	20.20± 0.09 ^{f}	28.22± 0.08 ^{g}	12.19± 0.09 ^{c}	4.42± 0.03 ^{b}	6.98± 0.05 ^{c}	27.17± 0.19 ^{j}	20.68± 0.14 ^{f}	29.07± 0.11 ^{h}	29.78± 0.19 ^{h}	12.34± 0.12 ^{b}	13.89± 0.14 ^{b}	65.84± 0.21 ^{h}
	**t=25 °C / preserved 7 days**	72.41± 0.19 ^{j}	71.09± 0.25 ^{i}	71.17± 0.20 ^{i}	10.19± 0.12 ^{c}	15.29± 0.15 ^{d}	8.47± 0.06 ^{b}	4.92± 0.03 ^{b}	6.40± 0.07 ^{c}	16.90± 0.14 ^{f}	11.32± 0.09 ^{c}	16.58± 0.13 ^{d,e}	18.90± 0.11 ^{e}	25.77± 0.11 ^{d}	22.71± 0.12 ^{d}	63.38± 0.09 ^{g}
	**t=38 °C / preserved 4 h**	71.48± 0.11 ^{i}	69.06± 0.13 ^{h}	71.08± 0.19 ^{i}	8.12± 0.14 ^{b}	13.54± 0.11 ^{d}	8.08± 0.08 ^{b}	2.39± 0.02 ^{a}	2.78± 0.03 ^{b}	16.76± 0.18 ^{f}	8.46± 0.07 ^{b}	13.82± 0.09 ^{d}	18.61± 0.13 ^{e}	16.40± 0.08 ^{c}	11.60± 0.10 ^{b}	64.26± 0.15 ^{h}
**MAE 180-6**	**t=4 °C / preserved 7 days**	63.62± 0.16 ^{d}	57.44± 0.09 ^{a}	67.74± 0.28 ^{g}	18.48± 0.04 ^{e}	40.61± 0.01 ^{j}	17.51± 0.05 ^{e}	6.65± 0.01 ^{c}	7.25± 0.08 ^{c}	33.70± 0.17 ^{k}	19.64± 0.06 ^{e}	41.25± 0.07 ^{k}	37.98± 0.13 ^{j}	19.79± 0.08 ^{c}	10.12± 0.07 ^{a}	62.54± 0.14 ^{g}
	**t=25 °C / preserved 7 days**	73.00± 0.25 ^{j}	65.30± 0.18 ^{e}	69.80± 0.23 ^{h}	9.48± 0.09 ^{b}	28.49± 0.07 ^{g}	8.78± 0.10 ^{b}	5.79± 0.07 ^{c}	7.10± 0.09 ^{c}	19.36± 0.12 ^{g}	11.11± 0.14 ^{c}	29.36± 0.13 ^{h}	21.25± 0.16 ^{f}	31.41± 0.15 ^{e}	13.99± 0.16 ^{b}	65.61± 0.14 ^{h}
	**t=38 °C / preserved 4 h**	71.66± 0.30 ^{i}	63.20± 0.27 ^{d}	72.74± 0.22 ^{j}	5.01± 0.08 ^{a}	22.74± 0.04 ^{f}	7.07± 0.06 ^{b}	8.14± 0.12 ^{d}	4.98± 0.04 ^{b}	18.57± 0.27 ^{f,g}	9.56± 0.13 ^{b}	23.28± 0.12 ^{f,g}	19.87± 0.18 ^{e,f}	58.39± 0.15 ^{f}	12.35± 0.14 ^{b}	69.16± 0.20 ^{h,i}
**MAE 300-6**	**t=4 °C / preserved 7 days**	60.26± 0.19 ^{c}	59.19± 0.16 ^{b,c}	71.24± 0.21 ^{i}	15.38± 0.05 ^{c}	39.05± 0.09 ^{i}	9.92± 0.04 ^{b}	7.06± 0.03 ^{c}	7.47± 0.07 ^{c}	21.44± 0.25 ^{g,h}	23.76± 0.09 ^{g}	39.76± 0.11 ^{j}	23.62± 0.17 ^{g}	24.66± 0.11 ^{d}	10.83± 0.09 ^{b}	65.17± 0.19 ^{h}
	**t=25 °C / preserved 7 days**	69.53± 0.11 ^{h}	59.64± 0.26 ^{c}	67.35± 0.14 ^{g}	7.50± 0.09 ^{b}	37.91± 0.18 ^{i}	12.24± 0.09 ^{c}	4.48± 0.06 ^{b}	10.45± 0.05 ^{d}	24.27± 0.21 ^{i}	8.74± 0.08 ^{b}	39.32± 0.16 ^{j}	27.18± 0.14 ^{h}	30.85± 0.06 ^{e}	15.41± 0.15 ^{b}	63.24± 0.17 ^{g}
	**t=38 °C / preserved 4 h**	72.65± 0.13 ^{j}	58.98± 0.17 ^{b}	69.35± 0.22 ^{h}	11.54± 0.10 ^{c}	30.57± 0.14 ^{h}	9.47± 0.19 ^{b}	1.99± 0.02 ^{a}	5.41± 0.03 ^{c}	25.88± 0.28 ^{i}	11.71± 0.08 ^{c}	31.05± 0.11 ^{i}	27.56± 0.24 ^{g,h}	9.78± 0.10 ^{a}	10.04± 0.07 ^{a}	69.90± 0.28 ^{i}

FJ' - frozen jostaberry; FDJ' – freeze-dried jostaberry; DJ' – oven dried jostaberry, obtained in optimal condition. L* - lightness; a* - red-green parameter; b* - yellow-blue parameter; C* - chromaticity; h* - hue angle. UAE 20- jostaberry extract obtained by ultrasound assisted extraction 20 minutes; MAE 100-6 – jostaberry extract obtained by microwave assisted extraction, magnetron power 100 W, 6 minutes; MAE 180-6 – jostaberry extract obtained by microwave assisted extraction, magnetron power 180 W, 6 minutes; MAE 300-6 – jostaberry extract obtained by microwave assisted extraction, magnetron power 300 W, 6 minutes. The results are presented as the mean of three measurements ± standard deviation (SD). Different letters ([a–k]) designate statistically different results ($p \leq 0.05$)

Table S5. The influence of pH and storage conditions on AA (DPPH, ABTS) and color parameters (L*, a*, b*, C* and h*) of jostaberry extracts obtained under optimal conditions with the application of UAE and MAE (FJ', FDJ', DJ')

Parameter	Influence of medium pH, bits		Influence of storage condition, bits	
	**UAE**	**MAE**	**UAE**	**MAE**
Antioxidant activity (DPPH)	0.389	0.426	0.111	0.352
Antioxidant activity (ABTS)	0.499	0.315	0.278	0.204
Lightness, L*	0.055	0.241	0.055	0.111
Red-green parameter, a*	0.167	0.241	0.222	0.259
Yellow-blue parameter, b*	0.278	0.203	0.167	0.204
Chromaticity, C*	0.333	0.204	0.222	0.278
Hue angle, h*	0.278	0.130	0.167	0.111

UAE - ultrasound assisted extraction; MAE - microwave assisted extraction.
